# Distinct feedforward and feedback pathways for cell-type specific attention effects

**DOI:** 10.1016/j.neuron.2024.04.020

**Published:** 2024-05-16

**Authors:** Georgios Spyropoulos, Marius Schneider, Jochem van Kempen, Marc Alwin Gieselmann, Alexander Thiele, Martin Vinck

**Affiliations:** 1 Ernst Strüngmann Institute (ESI) for Neuroscience in Cooperation with Max Planck Society, 60528 Frankfurt am Main, Germany; 2 Donders Centre for Neuroscience, Department of Neuroinformatics, Radboud University Nijmegen, 6525 Nijmegen, the Netherlands; 3 Biosciences Institute, Newcastle University, Newcastle upon Tyne NE1 7RU, UK

## Abstract

Selective attention is thought to depend on enhanced firing activity in extrastriate areas. Theories suggest that this enhancement depends on selective inter-areal communication via gamma (30–80 Hz) phase-locking. To test this, we simultaneously recorded from different cell types and cortical layers of macaque V1 and V4. We find that while V1-V4 gamma phase-locking between local field potentials increases with attention, the V1 gamma rhythm does not engage V4 excitatory-neurons, but only fast-spiking interneurons in L4 of V4. By contrast, attention enhances V4 spike-rates in both excitatory and inhibitory cells, most strongly in L2/3. The rate increase in L2/3 of V4 precedes V1 in time. These findings suggest enhanced signal transmission with attention does not depend on inter-areal gamma phase-locking and show that the endogenous gamma rhythm has cell-type- and layer-specific effects on downstream target areas. Similar findings were made in the mouse visual system, based on opto-tagging of identified interneurons.

## Introduction

Attention reflects the ability to selectively process and respond to behaviorally relevant sensory information. Higher mammals like primates have the capacity to direct their attention toward specific stimuli based on learned cue-reward associations. The modulation of neural stimulus responses caused by top-down attention is thought to be mediated by cortical feedback from frontal areas (e.g., FEF) toward sensory areas (e.g., V4).^1–3^ An outstanding question is the nature of the circuit mechanisms through which top-down feedback selectively enhances the sensory processing of attended sensory stimuli.

To answer this question, three major challenges need to be overcome: first, selective attention modulates distinct aspects of neural activity, and does so in multiple cortical areas simultaneously. One consistent finding is the enhancement of stimulus-evoked spike rates, especially in higher levels of the primate ventral stream.^4^ This increase in spike rates can be understood as a gain modulation of firing responses to the attended stimulus.^5^ Another main finding is an increase in inter-areal phase-locking in the gamma-frequency (30–80 Hz) range between areas.^6–8^ In order to formulate a complete mechanistic account of attention, these two phenomena must be put together in a coherent way. Second, a profound challenge in the dissection of circuit mechanisms is the interwoven nature of feedback and feedforward connections due to the reciprocal connections between cortical areas. For instance, it is possible that feedback from, e.g., frontal areas induces changes in V1 activity that lead to subsequent changes in neural activity in downstream areas like V2 and V4.^9^ However, it is also possible that feedback modulates activity in, e.g., area V4, which, in turn, modulates activity in area V1. This influence from V4 to V1 may, then, lead to subsequent feedforward changes in area V4, thereby closing a functional loop.^4,10^ Crucially, feedforward and feedback connections are organized along cortical layers, which therefore enable the separation of information flow related to feedforward and feedback processing.^11,12^ Third, the circuit mechanisms of attention likely rely on specific interactions between GABAergic interneurons and excitatory neurons, which are difficult to target, particularly in primates. Previous work has suggested differences in attentional modulation between these cell types^13–15^; however, it remains to be determined whether they show specific modulations related to inter-areal interactions and feedback/feedforward processing. For instance, feedforward projections tend to predominantly target excitatory cells and fast-spiking interneurons in the granular layer, whereas feedback targets excitatory cells and a broader set of different interneuronal classes.^12,16,17^

There are two main competing theories of the mechanisms underlying selective attention that have made specific proposals about the nature of feedforward and feedback interactions mediating attentional modulation. According to one major hypothesis, attentional selection leads, via top-down feedback, to the enhancement of feedforward (FF) inter-areal information transmission via inter-areal oscillatory synchronization.^9,18,19^ Enhanced effective communication is then thought to induce subsequent increases in neural responses (i.e., firing rates) for attended stimuli downstream.^9,18,19^ A competing hypothesis posits that the main effect of selective attention is to enhance the gain of neural responses for attended stimuli via top-down feedback (FB), with the strongest and earliest effects at higher hierarchical levels closer to behavioral responses.^1,4,20,21^ Such gain modulation may depend on, e.g., the top-down modulation of specific GABAergic interneurons, like SSt+ or VIP+ interneurons.^22^ These two hypotheses (rate vs. phase-locking) are not necessarily mutually exclusive, and it is possible that distinct roles are played by these two mechanisms considering the laminar organization of feedforward and feedback connections.^12^ For instance, enhanced feedforward processing via gamma-synchronization may primarily lead to changes in neural activity in the granular layer (L4) of downstream areas, whereas feedback may primarily modulate neural activity in the extra-granular layers.

A major impediment to testing these theories and determining the nature of feedforward-feedback interactions is the lack of simultaneous recordings from multiple areas of the primate cortex at a laminar and cell-type specific resolution. Using laminar probes, we recorded local field potentials (LFPs) and well-isolated single units from areas V1 and V4 in macaque monkeys performing a spatial attention task. We then analyzed the way in which distinct cell classes in area V4, across different cortical laminae, were phase-locked to the V1 gamma-rhythm, and how this long-range phase-locking was modulated by attention. We contrasted these effects with the cell- and layer-specific attentional modulation of stimulus-driven firing rates and performed population decoding analyses to determine which signals tracked attention reliably from trial to trial. Finally, we analyzed Neuropixels data from mice and investigated the inter-areal phase-locking of different GABAergic subtypes, which were identified through optogenetic tagging.

## Results

**Figure 1:**
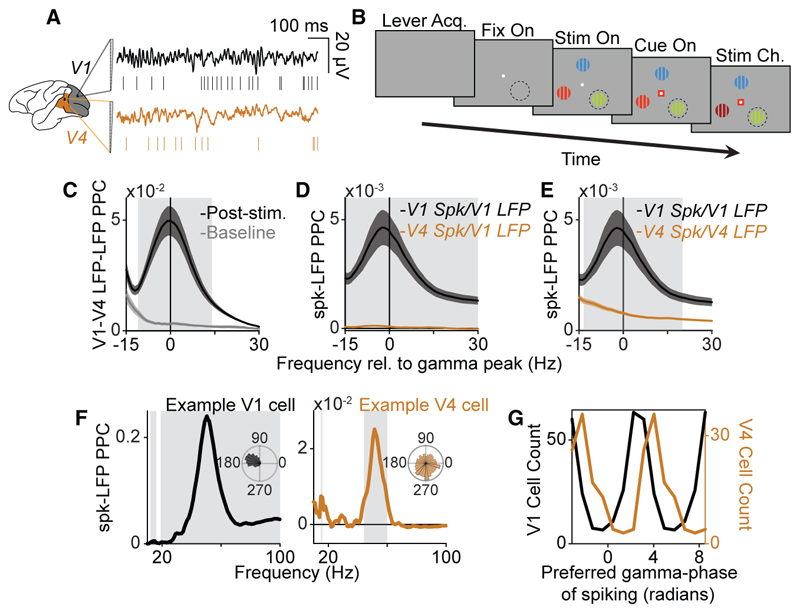
Feedforward gamma-band synchronization between V1 and V4 in the monkey (A) Experimental setup in the macaque. (B) Illustration of selective attention task. The dotted black circles illustrate the RFs of V1 and V4 neurons (i.e., these dotted black circles were not presented on the screen). (C) Phase-locking (pairwise-phase-consistency, PPC; see STAR Methods) between V1 and V4 LFPs during the baseline and the period between stimuli-onset and the last stimulus change (*n* = 68 sessions). Note that the data used for the spectra include trials of both attention conditions. (D) PPC between V1 LFPs and V1 single units (SU) (*n* = 311) or V4 SUs (*n* = 397). (E) Same as Figure 1D, but for V1 LFPs and V1 SU spiking, and V4 LFPs and V4 SU spiking. (F) Phase-locking spectra of V1 LFPs and the spiking of an example SU in V1 and V4. Insets show the average phase of each spike across V1 LFP channels. Gray rectangles designate significantly different frequency bins, Rayleigh’s test for uniformity, Bonferonni correction for multiple comparisons with a threshold of *p* < 0.05). (G) Mean V1-gamma phase of spiking for significantly gamma-locked V1 or V4 SUs (mean phase difference = 1.51 radians, *p* = 0, randomization test). (C–E) Confidence intervals designate SEM across cells, gray rectangles designate significantly different frequency bins, randomization test between cell types, FDR correction for multiple comparisons with a threshold of *p* < 0.05).

We recorded laminar local field potentials (LFPs) and single-unit spiking-activity simultaneously from areas V1 and V4 (Figure 1A), while macaques performed an attention task (Figure 1B; see STAR Methods and van Kempen et al.^23^ for more details). Each trial in the task started when the macaques grabbed a lever and foveated a central fixation spot. Releasing the lever or breaking fixation led to the immediate termination of the trial. Subsequently, three colored gratings appeared in the visual periphery. One of these three gratings was centered on the receptive fields of the recorded neural populations in both V1 and V4. After the onset of the stimuli, a colored cue, surrounding the fixation spot, directed attention to the peripheral grating that matched the cue’s color. This target stimulus was selected pseudo-randomly in each trial. The macaques received a liquid reward after releasing the lever in response to a change in the target stimulus, while ignoring potential changes in the other peripheral gratings.

### Feedforward propagation of V1 gamma rhythm

**Figure 2:**
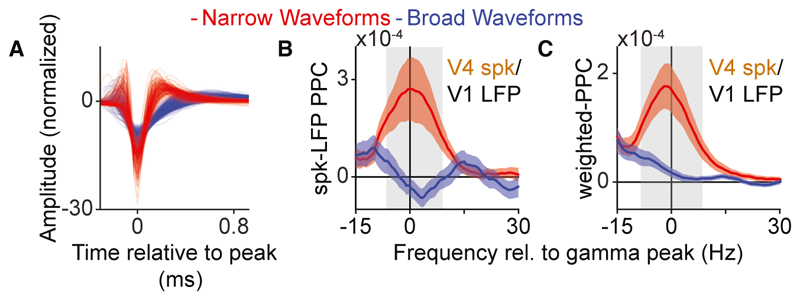
Feedforward gamma-band syn-chronization between V1 and V4 in the monkey mainly engages downstream interneurons (A) Normalized spike waveforms of V4 SUs. (B) Only NW V4 neurons show gamma phase-locking to V1 LFPs (NW: *n* = 152; BW: *n* = 216). (C) same as (B), but after weighting the PPC spectrum corresponding to each cell by the cell’s number of spikes. (B and C) Confidence intervals designate SEM across cells, gray rectangles designate significantly different frequency bins, randomization test between cell-types, FDR correction for multiple comparisons with a threshold of *p* < 0.05).

Consistent with previous studies in macaques,^6,7^ visual stimulation increased gamma-band phase-locking between V1 and V4 LFPs (Figure 1C). Several further analyses indicated that V1-LFP-V4-LFP gamma phase-locking did not result from coupling of intrinsic V1 and V4 oscillations, but from the feedforward propagation of the V1 gamma rhythm: (1) LFP-LFP locking was accompanied by only weak V4-spike-to-V1-LFP locking, especially when compared to local V1-spike-to-V1-LFP locking (Figures 1D and 1F); (2) V4-spike-to-V4-LFP locking did not display a clear peak in the gamma range (Figure 1E); and (3) on average, V1 cells spiked at earlier V1 gamma phases than V4 cells (mean phase difference = 1.51 radians) (Figures 1F and 1G). We note that these results are consistent with the Granger-causality analyses on the same dataset shown by Ferro et al.,^8^ which shows feedforward Granger-causal influences from V1 LFPs to V4 LFPs in the gamma frequency-band.

### Cell-type specific phase-locking of V4 neurons to V1 gamma rhythm

To gain deeper physiological insight into these observations, we analyzed the waveform characteristics of V4 single-unit spiking. Units could be clearly separated into two main classes with broad (BW) and narrow (NW) waveforms (Figure 2A) corresponding to putative excitatory and fast-spiking (FS) interneurons, respectively^13,14,24,25^ (but see Vigneswaran et al. and Dasilva et al.^26,27^). In agreement with previous findings,^13,14^ BW cells had lower firing-rates than NW cells (baseline: mean BW FR = 2.5564, mean NW FR = 3.4101, *p* = 0.0153; visual stimulation: mean BW FR = 4.7587, mean NW FR = 9.3089, *p* = 1.303 × 10^−6^; randomization test between cell types). Next, we analyzed the long-range phase-locking between V4 cell types and the V1 gamma rhythm. Surprisingly, only NW neurons showed a gamma-frequency peak in the V4-spike-to-V1-LFP phase-locking spectrum (Figure 2B). This difference was not explained by the lower spiking rates of BW cells, because we used a metric that is unbiased by firing rate, and the difference was also observed when phase-locking spectra were weighted by the number of spikes of each cell^14^ (Figure 2C). The strength of spike-field locking was independent of stimulus drive for BW cells, and only weakly correlated with stimulus drive for NW cells (Figure S2A).

### Attentional modulation of V4-spike-to-V1 phase-locking and firing rates

**Figure 3:**
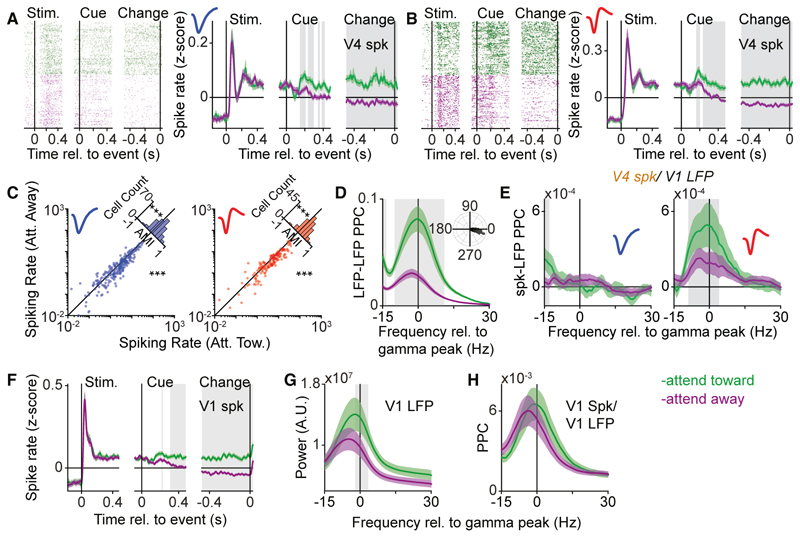
The cell-type specific effect of attention on gamma-band synchronization between V1 LFPs and V4 cells in the monkey (A) Left: Example V4 BW cell. Right: Average peri-event time histogram (PETH) across V4 BW cells (*n* = 240). (B) Same as (A), but for V4 NW cells (*n* = 164). (C) Mean firing rates for the attend-toward and attend-away conditions. Insets: Attentional Modulation Index (AMI). ***p < 0.001; randomization test between attentional conditions, Wilcoxon’s signed-rank test across cells. (D) Phase locking (PPC) between V1 and V4 LFPs, for the two attention conditions during the period after cue onset. Inset: Relative phase difference between attention conditions across all V1 and V4 LFP pairs (mean angle = −0.098 radians). (E) Phase-locking (PPC) between V1 LFPs and V4 BW (*n* = 162) and NW (*n* = 134) spiking. (F) Same as (A) (right) but for V1 SUs (*n* = 335). (G) Mean power of V1 LFPs for the two attentional conditions. (H) Same as (E) but for V1 LFPs and V1 SU spiking (*n* = 254). (A,B,D-H) Confidence intervals designate SEM across cells (A,B,E,F,H) or sessions (D,G, *n* = 68). Gray rectangles designate significantly different time or frequency bins, randomization test between cells or sessions, FDR correction for multiple comparisons with a threshold of *p* < 0.05).

Together, these analyses show that long-range V1-V4 gamma-synchronization is highly cell type specific. We wondered if the attentional modulation of V4-spike-to-V1-LFP phase-locking would therefore also be cell type specific. Furthermore, if the attentional modulation of V4 firing rates depends on an increase in V1-to-V4 signal transmission, then this would predict a differential attentional rate modulation of V4 NW and BW neurons. In contrast to this prediction, we found that the firing rates of both BW and NW neurons showed a comparable increase with attention (Figures 3A–3C). The increase in spiking with attention for BW neurons was observed even though the V4-spike-to-V1-LFP phase-locking spectra of BW cells did not display gamma peaks in either attentional condition (Figure 3E, left).

Despite this lack of attentional modulation, there was a robust increase in gamma-band phase-locking between V1 and V4 LFPs, for the period after cue-onset, in accordance with previous reports^6,7^ (Figure 3D). However, attention-related changes in gamma-band phase-locking of LFPs arose substantially later than changes in V4 spiking rates (Figure S3). Different from V4 BW neurons, we found that NW cells displayed stronger spike-to-V1-LFP gamma-band phase-locking with attention (Figure 3E, right). This difference between NW and BW neurons was not due to a potential signal-to-noise ratio discrepancy related to differences in spiking rates in the two attention conditions (Figure S4). Furthermore, the lack of attentional modulation of V4-to-V1 phase-locking in BW neurons (or even the complete absence of phase-locking) was also observed for BW neurons with a strong attentional modulation (Figures S2B and S2C).

The increase in V4-to-V1 phase-locking with attention observed for V4 NW-neurons and V4 LFPs was likely partially due to changes in activity in V1,^28,29^ including an increase in firing rates (Figure 3F), gamma power, and peak frequency (Figures 3G and 3H). Yet, the increased propagation of the V1 gamma rhythm to V4 only affected putative FS neurons, which is likely due to the difference in filtering properties between FS and excitatory neurons.^30,31^

### Decoding of attentional state: Gamma phase-locking versus firing rates

**Figure 4:**
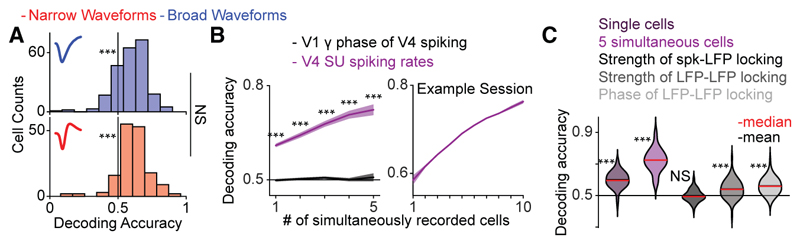
Spiking rates are more reliable in encoding the attentional state compared to measures of inter-areal gamma-band synchronization (A) Decoding accuracy of the attentional condition based on the firing rates of BW (*n* = 231) and NW cells (*n* = 160). (B) Left: Decoding accuracy of the attentional condition as a function of the number of simultaneously recorded cells for V4 SU spiking rates or the V1 gamma phase of V4 spiking, across sessions. Right: Decoding accuracy based on firing rates for an example session. Confidence intervals designate SEM across sessions (B [left], *n* = 68) or cells (B [right]). (C) Violin plots of the distribution of decoding accuracy across sessions for decoding based on different modalities (black lines representing the mean are not clearly visible because they greatly overlap with red lines representing the median). Modalities related to synchronization are the strength of phase-locking of V4 SU spikes to V1 gamma-band LFPs, the strength of phase-locking between gamma-band LFPs in V1 and V4, and the relative phase of gamma-band LFPs in V1 and V4. (A–C) ***p < 0.001; Wilcoxon’s signed-rank test across cells (A) and sessions (C, *n* = 68), randomization test between cell-types (A) or across sessions (B [left]).

We further wondered how reliably the attentional state was encoded by firing rates and gamma phase-locking, respectively, by performing decoding analyses of the attentional state separately on spiking rates or various measures of inter-areal gamma-band synchronization. The accuracy of decoding based on V4 single-cell spiking rates was significantly above the chance level, with both cell types displaying similar accuracy (Figure 4A). For this reason, we pooled cell types for our subsequent decoding analyses, which were based on population spiking rates. Decoding accuracy increased monotonically as a function of the number of simultaneously recorded neurons, without reaching an asymptote for 5 or even 10 cells (Figure 4B). This suggests that the upper bound of information related to the attentional state, as encoded in V4 population spiking, far exceeds what can be gleaned in our findings. By contrast, decoding based on the V1 gamma-phase of V4 spiking (Figure 4B) or the strength of spike-LFP phase locking in the gamma range (Figure 4C) were indistinguishable from chance. The relative gamma phases of V1 and V4 LFP pairs and the strength of their phase-locking in the gamma range were significantly more informative than chance, but still less informative than even single cell spiking in V4 (*p* = 3.615 × 10^−8^ for LFP-LFP phase-locking strength and *p* = 2.382 × 10^−5^ for the relative phase of LFPs, randomization test across sessions; Figure 4C).

To further investigate to what extent firing rates and gamma-synchronization provided independent information about the attentional state, we analyzed the relationship between the trial-by-trial decoding error of SU spiking and different measures of synchronization. We did not detect a consistent relationship between these variables across cells (Figure S5).

### Laminar distribution of attentional rate modulation and gamma phase-locking

**Figure 5:**
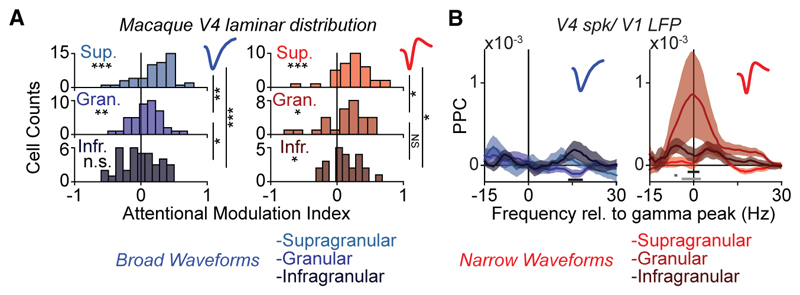
Inter-areal gamma-synchronization and attention in macaques involve cells in different laminar compartments (A) AMIs at different laminar compartments for BW (left) and NW cells (right) in macaque V4. *p < 0.05, **p < 0.01, ***p < 0.001; Wilcoxon’s signed-rank test across cells and randomization test between laminar compartments (BW: Nsup. = 52, Ngra. = 42, Ninf. = 37, NW: Nsup. = 34, Ngra. = 30, Ninf. = 23). (B) PPC between V1 LFPs and V4 cell spiking in different laminar compartments for BW (left) and NW (right) cells (BW: Nsup.= 43, Ngra. = 39, Ninf.= 34, NW: Nsup.= 30, Ngra. = 26, Ninf. = 23). This analysis included trials corresponding to both attentional conditions. (A and B) Bright, intermediate, and dark colors designate cells in superficial, granular, and deep layers, respectively. (B) Bright, intermediate, and dark horizontal bars in PPC spectra designate significantly different frequency bins between the superficial and granular compartment, the superficial and infragranular compartment, and the granular and infragranular compartment, respectively. Statistical comparisons: Randomization test between cell types, FDR correction for multiple comparisons with a threshold of *p* < 0.05.

The analyses above suggest that the V1-to-V4 gamma-band phase-locking results from the feedforward propagation of the V1 gamma rhythm. This would predict that gamma phase-locking should be specific to the granular input layer of area V4. Indeed, consistent with the FF propagation of the V1 gamma-rhythm, the phase-locking of V4 cells to V1 gamma-range LFPs was strongest in the granular input layer and only clearly present for NW cells, suggesting that V4-to-V1 gamma-phase locking did not propagate to the superficial layers of V4 (Figure 5B). By contrast, the strength of this attentional modulation decreased with depth for the spiking rate of BW cells, whereas it was more uniform across layers for NW cells (Figure 5A). For both cell types, we observed the strongest effects of attention in the spiking of neurons in superficial layers. This finding was not a product of potential differences in the spiking propensity of cells in different laminar compartments, as shown by a multi-linear regression analysis (Table S1).

In addition, we examined if there is a relationship between the attentional modulation of firing rates and the attentional modulation of feedforward gamma-rhythmic synchronization, separately for the two cell types and laminar compartments. We found that these variables were uncorrelated for both cell types in the superficial and granular layers of V4 (Figure S6).

### Latency of attentional effects on spike rates and spike-rate correlations

**Figure 6:**
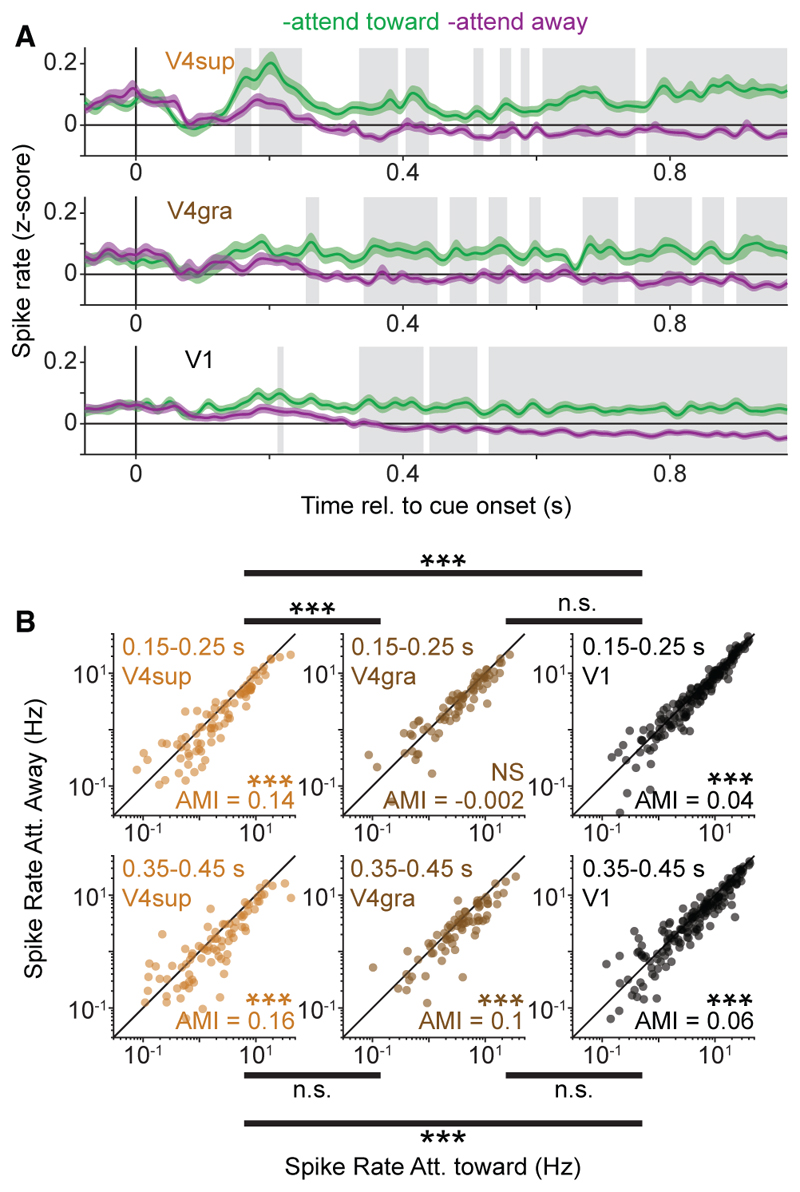
Superficial layers in V4 exhibit shorter attentional latencies in spiking compared to the input layer and V1 (A) Average PETH across SUs in superficial layers of V4 (top), the input layer in V4 (middle), and in V1 (bottom) for the two attentional conditions. Spiking activity is triggered around cue onset. Statistical comparisons were performed in the same manner as Figure 3A (right). (B) Mean spiking rate of SUs in superficial layers of V4, the input layer in V4, and in V1 for the attend-toward (abscissa) and attend-away conditions (ordinate). Spiking rates were computed for the period between 0.15 and 0.25 s after cue onset (top row) and the period between 0.35 and 0.45 s after cues onset (bottom row). Randomization test between compartments; ***p < 0.001. A randomization test across cells was also used to assess significance of AMIs for a given population/period; ***p < 0.001. (A and B) NV4sup. = 90, NV4gra. = 80, NV1. = 181.

The absence of a clear relationship between V1-V4 gamma phase-locking and the attentional modulation of V4 firing-rates shown in these analyses indicates that V4 rate changes likely result from a different mechanism than V1-V4 gamma phase-locking. Above, we reported that there was a stronger attentional modulation of firing-rates in the superficial layers as compared to the granular input layer of V4 (Figure 5). One possibility is that these rates changes directly result from top-down feedback to area V4.^2,3,10,20,32^ This scenario may predict that the attentional modulation of superficial V4 units precedes the modulation of V1 firing rates within a trial, as suggested by previous work using multi-unit analyses.^4^ To investigate this, we performed several analyses in which we examined the timing of attentional modulation relative to the attentional cue onset. We find that V4 superficial units showed, on average, a significant attentional modulation early after the attentional cue onset, while this was not the case for V4 granular units (Figure 6B). Firing rates showed significant changes with attention relatively early in superficial layers of V4 compared to V4 granular units and V1 units (Figure 6A). To further compare the timing of attentional effects between processing stages, we restricted the analysis to significantly modulated neurons, quantified the attentional modulation as a function of time, and computed a cross-correlation function based on these time courses. This analysis shows that the attentional modulation in V4 exceeds the modulation in V1 at early latencies and that the cross-correlation function showed an asymmetry with V4 leading V1 (Figure S7). These findings suggest that firing rate increases in superficial layers of V4 are at least partially independent of rate increases in V1.

We further investigated how attention modulated firing rate correlations at longer time scales compared to the gamma cycle. To investigate this, we examined time-resolved firing correlations between V1 and V4, and within V1 and V4 (using windows of ±40 ms, slid every 10 ms). We observed a substantial decrease in the magnitude of both intra- and inter-areal firing-rate correlations with attention (Figure S8). Changes in correlated firing were observed at relatively long latencies (after around 500 ms following the cue onset) and the decrease in correlations became larger toward the stimulus change (Figure S8A). We further quantified noise correlations in two different periods, namely the (early) 0.2–0.7 s period after the cue and the (late) 500 ms before the target change. In the 0.2–0.7 s period after the attentional cue onset, the decrease in noise correlations was significant only within V1 and between V1 and V4 granular units, but not between V1 and V4 superficial units or within V4 (Figure S8B, left; Figure S9B, left). In the late trial period, i.e., 500 ms before the target change, correlations were reduced within and between all areas/compartments (Figure S8B, right; Figure S9B, right). Analyses of directed interactions via Granger-causality showed a decreased influence of V1 onto V4 with attention (Figure S10; Table S2).

### Cell-type specific phase locking in mice

**Figure 7:**
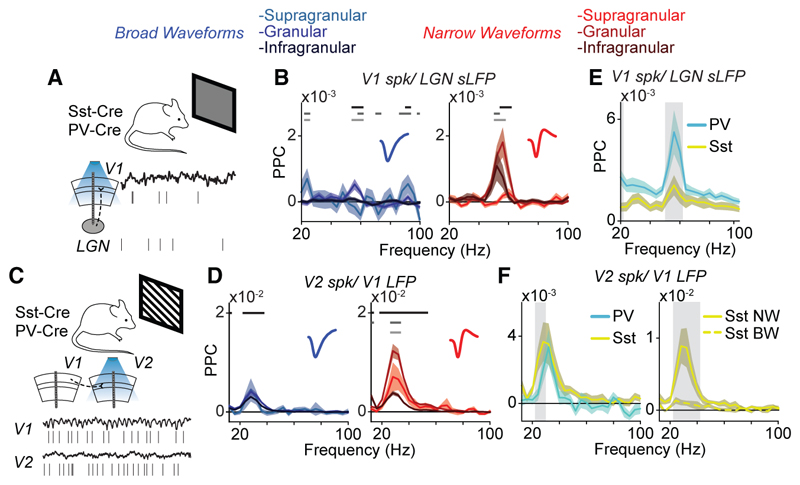
Inter-areal gamma-synchronization in mice mainly involves FS interneurons in feedforward input layers (A) Experimental setup in mice for the luminance condition. (B) Same as Figure 5B, but for V1 SUs and LGN spike-derived LFPs (sLFP) under the luminance-gamma condition, in mice (BW: Nsup. = 148, Ngra. = 463, Ninf. = 1055, NW: Nsup. = 47, Ngra. = 226, Ninf. = 316). (C) Experimental setup in mice for the contrast condition. (D) Same as B, but for V2 SUs and V1 LFPs under the contrast-gamma condition, in mice (BW: Nsup. = 23, Ngra. = 428, Ninf. = 2187, NW: Nsup. = 11, Ngra. = 281, Ninf. = 544). (E) PPC between LGN sLFPs and V1 cell spiking under the luminance-gamma condition, for PV+ (*n* = 47) or Sst+ cell spiking (*n* = 30). (F) Left: PPC between V1 LFPs and V2 cell spiking under the grating-gamma condition for PV+ (*n* = 32) or Sst+ cells (*n* = 21). Right: PPC between V2 LFPs and V1 Sst+ cell spiking under the grating-gamma condition, for BW (*n* = 14) or NW cells (*n* = 7). (B and D) Bright, intermediate, and dark colors designate cells in superficial, granular, and deep layers, respectively. Bright, intermediate, and dark horizontal bars in PPC spectra designate significantly different frequency bins between the superficial and granular compartment, the superficial and infragranular compartment, and the granular and infragranular compartment, respectively. (B and D–F) Statistical comparisons: Randomization test between cell types, FDR correction for multiple comparisons with a threshold of *p* < 0.05.

To physiologically characterize interneurons via optogenetic tagging and to investigate whether our laminar results are generalizable, we analyzed simultaneous recordings in the lateral geniculate nucleus (LGN) and visual cortical areas V1 and V2 (VISl, VISal, VISrl, VISam, and VISpm) of awake mice under conditions of visual stimulation (Figures 7A and 7C) (experiments were performed by the Allen Institute for Brain Science). We compared two conditions of passive visual stimulation during active states (i.e., high arousal and locomotion): (1) In the “luminance” condition, mice viewed a gray screen, which gives rise to a faster gamma rhythm (~60 Hz) generated in the LGN (Figure S11A; see also Schneider et al. and Saleem et al.^28,33^; (2) in the “contrast” condition, mice viewed drifting gratings, which give rise to a slower gamma rhythm (~30 Hz), thought to be generated in the superficial compartment of V1 (Figures S11B and S13B; see also Veit et al.^34^).

Our findings in mice showed a close similarity to our observations in the macaque: (1) The strength of phase-locking of spikes to the gamma generator (LGN and V1 respectively for the two conditions) was relatively weak in post-synaptic targets (V1 and V2, respectively; Figures S11A and S11B); (2) spiking in the respective gamma generator occurred at an earlier gamma phase compared to the spiking of its post-synaptic targets (Figures S11C and S11D); (3) both the LGN and V1 gamma preferentially engaged NW cells in the downstream area (Figure S12A, for waveforms in V1 and V2), independent of brain state (Figures S12B–S12G); (4) the strength of this feedforward gamma-rhythmic synchronization was, at most, very weakly dependent on stimulus selectivity for both BW and NW cells (Figures S2D–S2F); (5) phase-locking between the gamma generator (LGN and V1, respectively) and spiking in downstream cells (V1 and V2, respectively) was concentrated in FF input-layer and strongest for NW cells (Figure 7B for luminance gamma; Figure 7D for contrast gamma).

We investigated the differential contribution of interneuronal subtypes to inter-areal gamma synchronization via the optogenetic tagging of units corresponding to PV+ and Sst+ interneurons in mouse V1 and V2 (Pvalb-IRES-Cre and Sst-IRES-Cre lines, respectively). This analysis revealed that LGN gamma in the luminance condition predominantly engaged V1 PV+ cells (Figure 7E), the majority of which displayed an NW phenotype (Figures S14A and S14B). Surprisingly, both PV+ and Sst+ cells in V2 displayed phase locking with the V1 gamma rhythm under the contrast condition. However, a closer examination of the waveform characteristics of Sst+ cells revealed that NW, and not BW, Sst+ cells were preferentially locked to V1, suggesting that they display a fast-spiking phenotype^35^ (Figure 7F). These NW Sst+ cells were localized to the granular and infragranular layers, but not to the superficial layers (Figure S14C).

## Discussion

Attentional selection is thought to depend on the enhancement of stimulus-induced responses in extrastriate areas like V4. Theories suggest that enhanced V4 responses result from increased V1-to-V4 communication via 30–80 Hz gamma phase-locking.^9^ We tested this by determining the impact of the V1 gamma rhythm on different cell types across cortical layers of V4 in macaques. Similar to previous work,^6–8^ we observed that attention increased V1-V4 LFP gamma phase-locking. Yet, we showed that V1-V4 gamma phase-locking at the level of single units was highly cell-type- and layer-specific. Namely, we demonstrate a specific functional consequence of the afferent V1 gamma rhythm in driving L4 fast-spiking interneurons in the downstream target area V4. Our analyses in the mouse visual system revealed a similar pattern that was specific to optogenetically identified fast-spiking PV+ and narrow-waveform SSt+ interneurons.

In macaques, we could further show that attention increases the phase-locking of V4 fast-spiking interneurons to V1 gamma, but not the phase-locking of V4 excitatory neurons. In contrast to gamma phase-locking, the attentional modulation of firing rates in V4 was expressed by both excitatory and inhibitory neurons and was strongest in L2/3 instead of in L4. We furthermore observed that firing rate changes were substantially more reliable in encoding the attentional state as compared to inter-areal phase-locking. Finally, we find that the increase in V4 superficial firing rates precedes the increase in V1.

### Cell-type specific inter-areal interactions

It is commonly assumed that the rhythmic synchronization of spiking discharges in a pre-synaptic population increases their impact on a post-synaptic target population.^36–39^ Here we tested this assumption directly and separated the impact on post-synaptic fast-spiking interneurons and excitatory neurons. Our findings demonstrate that afferent gamma rhythms drive L4 fast-spiking interneurons, but not excitatory neurons, in a downstream target population. The observed differences in phase-locking properties agree well with known differences in the filtering (resonance) properties of E and I cells.^30,31,40–42^ These observations also agree with findings made in rodent CA1,^43^ wherein gamma-rhythmic input from the entorhinal cortex and CA3 mainly drive fast-spiking interneurons in the stratum lacunosum-moleculare and the stratum radiatum, respectively. Our analyses of identified GABAergic interneurons in mice further elucidated the physiological character of the fast-spiking interneurons: High-frequency (~55 Hz) gamma-synchronization in the LGN recruited almost exclusively V1 PV+ interneurons, mainly in L4. Low-frequency (~25 Hz) gamma-synchronization in V1 led to phase-locking in V2 PV+ interneurons, but also narrow-waveform SSt+ interneurons. It is possible that this subset of SSt+ neurons corresponds to non-Martinotti cells. These neurons, located in L4 and L5, generally have action potentials with narrower widths, exhibit higher firing rates, and mostly project locally.^35,44^ Thus, the fast-spiking interneurons in V4 may comprise a mixture of PV+ and narrow-waveform SSt+ interneurons.

We conclude that feedforward gamma-synchronization primarily engages downstream inhibitory cells. In our view, this finding reflects the feedforward amplification of inhibition in the downstream area: rhythmic input with energy in higher frequencies increases the gain of pre-synaptic spikes onto FS interneurons, due to the resonance properties of the latter.^30,40,45^ The lack of propagation of V1 gamma-synchronization beyond L4 of V4 contradicts the common conception that inter-areal LFP phase-locking reflects synchronization between relatively large neural populations,^9,46^ considering that neurons in L4 do not make feedback projections. These findings match with recent modeling studies suggesting that the increase in V1-V4 gamma phase-locking can result from changes in V1 gamma power, frequency and firing rates,^28,29,47^ and can be explained from linear interaction models without inter-areal synchronization between oscillators.^28,29,48^

The observed lack of phase-locking of V4 excitatory neurons to V1 gamma suggests a different interpretation of inter-areal phase-locking than the communication-through-coherence hypothesis, in which gamma-synchronization is thought to facilitate communication to excitatory projection neurons.^9^ An interesting possibility is that gamma-rhythmic input enhances feedforward inhibition, which has been suggested as a mechanism to increase the signal-to-noise ratio of feedforward information transmission via divisive normalization mechanisms.^49–53^ A complementary role for feedforward gamma-synchronization and the concomitant feedforward inhibition has been demonstrated in the circuit comprising the entorhinal cortex, dentate gyrus, and area CA3. In this circuit, inhibitory feedback was shown to be important for pattern separation.^54^ It is possible that similar computations are facilitated in visual cortex via the modulation of inter-areal gamma synchronization by processes such as attention, which requires also the separation between multiple competing inputs.^20^

### Distinct contributions of gamma and firing rates to attention

The observation that attention modulates both firing rates and inter-areal phase-locking raises the question of their relative importance in the neural implementation of attention. To investigate this, we performed population decoding analyses and established that V4 firing rates are substantially more reliable than the phase-locking strength or the relative phase of V1 and V4 LFPs in encoding the locus of attention. This is the case even at the level of single cells, with a notable increase in reliability when considering multiple simultaneously recorded neurons. It is instructive to consider how a noisy signal (such as the firing rate of a single neuron) can be more reliable than a population-wide signal (such as LFPs in V1 and V4) in encoding a population-wide modulatory state such as attention. A modeling study by Akam et al.^55^ has shown that a modulation of phase-locking (increase for attended target) is unable to reliably achieve selective communication with a downstream receiving population. This is the case because input from a non-selected sender population will, by chance, often arrive at downstream phases of high excitability, even if the selected sender is perfectly phaselocked with the receiver.^48,55^ Our results on decoding agree well with these previous modeling results.^55^

### Dissociation of V4 firing rate modulation and V1-V4 gamma phase-locking

Several of our findings suggest that the attentional rate modulation in area V4 units does not result from V1-V4 gamma-band synchronization: (1) we observed major differences in the laminar distribution of attentional rate modulation and inter-areal phase-locking. In particular, attentional rate enhancement is strongest and earliest in superficial V4 layers, and not significant in the granular input layer at early time points. By contrast, gamma-synchronization of V4 units to V1 gamma was prominent in the granular input layer, rather than in superficial layers.

(2) We observed major differences in the cell-type specificity of attentional rate modulation and inter-areal phase locking. Attentional rate modulation was observed both in V4 excitatory and inhibitory neurons. By contrast, gamma phase-locking of V4 units to V1 gamma was observed in fast-spiking interneurons, but not in excitatory neurons. Furthermore, attention enhanced gamma phase-locking in the V4 fast-spiking interneurons, but not excitatory neurons. (3) Across neurons, attentional rate modulation was not correlated with the attentional modulation of gamma phase-locking. (4) Firing rate effects were substantially more reliable than phase locking in encoding the attentional state. This held true both when comparing single V4 neurons to LFP-LFP V1-V4 gamma-locking or when comparing the firing rates to the phase-locking of single V4 neurons. (5) We did not observe a correlation between the trial-by-trial decoding performance of firing rates and gamma phase locking. (6) Attention-dependent increases in V1-V4 gamma phase-locking became significant relatively late in the trial compared to increases in V4 firing rates.

Together, these findings suggest that the attentional modulation of V4 firing rates does not depend on V1-V4 gamma-band synchronization, but rather on other mechanisms. Previous work had already shown that detectable gamma oscillations are evoked only by subsets of natural visual stimuli, which implies that selective attention in the visual modality cannot generally depend on V1 gamma oscillations.^56,57^

Attentional mechanisms can be broadly divided into two classes: (1) first, top-down activity may modulate the activity in earlier visual areas (e.g., V1), leading to a propagation of attentional effects in higher areas (e.g., V4); (2) top-down influences may act directly on higher processing levels like V4 and thereby amplify the stimulus-induced response. We observed that attentional rate modulation in V4 occurs early in time and predominantly in the superficial layers. A plausible explanation is therefore that these rate increases result from top-down feedback into superficial layers from frontal and parietal areas^1–3,20,32^ or higher-order thalamus.^58^ This would match the anatomical finding that feedback projections are particularly prominent into superficial layers.^12,59–62^

The idea that attentional enhancements result from feedback into superficial layers matches well a recent study that manipulated top-down projections from area V4 to V1 using the optogenetic inactivation of projections from V4 to V1.^10^ The inactivation of V4-to-V1 feedback projections resulted in a substantial decrease of upstream attentional modulation particularly in the superficial layers of V1, suggesting that the attentional modulation in V4 is driving the attentional modulation in V1. This finding matches well with the strength and latency of attentional effects that we observed in V4 as compared to V1 and supports the conclusion that attentional effects in V4 are driven at least in part from feedback from higher areas.^2^ However, the study of Debes et al.^10^ also suggests that attentional modulation in V4 is further enhanced as a result of the attentional modulation in area V1, as optogenetic suppression of V4-to-V1 feedback projections led to a reduction in attentional rate modulation in area V4. The authors did not analyze the laminar distribution of these effects in V4. Based on our data, we would predict that within V4, it is the attentional modulation of the granular compartment that depends most strongly on the attentional modulation in area V1. Furthermore, we would predict that this dependence is expressed at longer latencies after stimulus onset compared to changes in superficial V4 units. It is, furthermore, important to note that our study and the Debes et al.^10^ study used very different experimental paradigms. In their study, attention was cued in blocks of either 64 or 128 trials, such that the monkeys did not have to switch their attentional allocation between trials via an attentional cue that changed from trial to trial. This may explain why the attentional effects were already present at very early latencies after stimulus onset in their study. By contrast, in our study attention was cued from trial to trial, which may explain why the attentional effects in area V1 were observed relatively late after the onset of an attentional cue that changed randomly from trial to trial. Hence, it is important to repeat studies like Debes et al.^10^ with optogenetic suppression of feedback in paradigms as used in the present study. Altogether, the evidence presented here, combined with the causal manipulations by Debes et al.,^10^ suggests that the attentional effects in area V4 are at least to some extent not inherited from attentional modulation of firing rates in area V1, especially at early latencies.

To further understand the interactions between V1 and V4, future work should also combine laminar and cell-type specific recordings with analyses of statistical correlation between population activities.^63–65^ For example, recent work suggests that there are specific sub-spaces in V1 population activity that are predictive of activity in higher visual areas, and such statistical approaches (which require concurrent measurements of relatively large neural ensembles) could shed further light on the recurrent interactions between V1 and V4.^63,64^ It is important to emphasize, however, that top-down influences can be modulatory and may not necessarily be expressed in enhanced spontaneous firing rate correlations, but could nonetheless enhance stimulus-driven firing rates. Furthermore, correlations at distinct frequencies may be differentially affected. For example, in the present work, we find enhanced correlations at gamma frequencies between V1 and V4 activity, but found a decrease in spontaneous (noise) correlations between V1 and V4 at slower timescales. In both cases, the change was most prominent between V1 and granular V4 units. This result differs from that found in a previous study, in which attention increased inter-areal noise correlations.^66^ It is possible that the reduction in V1-V4 noise correlations observed in our study reflects a decrease in correlated firing within V1 itself,^28^ which we observed as in many previous studies.^67–69^ This reduction in noise correlations may also be related to the negative relation of attention with theta and alpha oscillations in primary visual cortex,^70,71^ although this remains to be directly explored. Reductions in noise correlations have been proposed to improve the fidelity of sensory information processing with attention,^72^ and it is possible that a reduction in inter-areal noise correlations has a similar effect.

## Conclusion

These findings provide, to our knowledge, the first description of inter-areal interactions between distinct cell types residing in separate cortical layers while animals perform a cognitive (attention) task. Our analyses revealed a motif that we also confirmed in mice, namely the recruitment of fast-spiking interneurons in a downstream target area via gamma-synchronization. Future work should identify the precise biophysical mechanisms underlying the phenomenon, and further investigate the generality of this observation. Furthermore, our data indicate that inter-areal gamma phase-locking and rate modulation constitute distinct mechanisms for top-down attention. Altogether, these findings reveal distinct, cell-type specific feedforward and feedback pathways for the attentional modulation of inter-areal synchronization and spike rates, respectively.

## Star⋆Methods

Detailed methods are provided in the online version of this paper and include the following:

- KEY RESOURCES TABLE
- RESOURCE AVAILABILITY
  ○ Lead contact
  ○ Materials availability
  ○ Data and code availability
- EXPERIMENTAL MODEL AND STUDY PARTICIPANT DETAILS
- METHOD DETAILS
  ○ Behavioral paradigm: Macaques
  ○ Data acquisition: Macaques
  ○ Data acquisition: Mice
  ○ Analysis software

- QUANTIFICATION AND STATISTICAL ANALYSIS
  ○ Receptive field mapping: Macaques
  ○ Preprocessing: Macaques
  ○ Spike sorting: Macaques
  ○ Waveform classification
  ○ Spectral Analysis
  ○ Filtering for gamma-band phase extraction and trial-by-trial estimation of phase-locking strength
  ○ Quantification of Spike-LFP phase-locking: Macaques
  ○ Quantification of Spike-LFP phase-locking: Mice
  ○ Drivenness, attentional effect in spiking, and stimulus selectivity indices
  ○ Decoding analyses: Macaques
  ○ Trial-by-trial decoding error
  ○ Assignment of cortical layers
  ○ Multi-linear regression
  ○ Analysis of latency in attention effects
  ○ Computation of noise-correlation and Granger-causality
  ○ Testing for optogenetic response: Mice
  ○ State detection: Mice
  ○ Statistical testing

## Star⋆Methods

### Key Resources Table

**Table:** 

REAGENT or RESOURCE	SOURCE	IDENTIFIER
Deposited data		
Electrophysiological data	Thiele lab	G-Node: https://doi.gin.g-node.org/10.12751/g-node.b0mnn2
Visual Coding - Neuropixels Dataset	Allen Institute for Brain Science	https://portal.brain-map.org/circuits-behavior/visual-coding-neuropixels
Experimental models: Organisms/strains		
Rhesus Macaque (Macaca mulatta)	Medical Research Council Center for Macaques (MRC CFM)	N/A
Software and algorithms		
MATLAB	MathWorks	https://mathworks.com/
Fieldtrip	Open Source	https://www.fieldtriptoolbox.org/

### Resource Availability

#### Lead contact

Further information and requests for resources should be directed to and will be fulfilled by the lead contact, Martin Vinck (martin. vinck@esi-frankfurt.de).

#### Materials availability

This study did not generate new unique reagents.

#### Data and code availability

- The data that support the findings of this study are available from the lead contact M.V.
- Analyses in this study were done in MATLAB and used the FieldTrip toolbox.^73^
- Any additional information required to reanalyze the data reported in this paper is available from the lead contact upon request.

#### Experimental Model And Study Participant Details

Our study involved two male adult rhesus macaque monkeys (Macaca mulatta, age 10–12 years, weight 8.5–12.5 kg), implanted with a head post and recording chambers over areas V1 and V4 under sterile conditions and general anesthesia. Housing conditions, surgical procedures, and post-operative care conditions have been described in considerable detail in previous papers^74,75^ and were in accordance with the UK Animals Scientific Procedures Act, the National Institute of Health’s Guidelines for the Care and Use of Animals for Experimental Procedures, and the European Communities Council Directive RL 2010/63/EC. Analyses in mice were based on the publicly available Visual Coding Neuropixels dataset, which was collected and preprocessed by the Allen Institute for Brain Science.^76^

#### Method Details

##### Behavioral paradigm: Macaques

Stimulus presentation and behavioral control were performed by using the Remote Cortex 5.95 software (Laboratory of Neuropsychology, National Institute for Mental Health, Bethesda, MD). We presented visual stimuli on a cathode ray tube (CRT) monitor, with a refresh rate of 120 Hz, a resolution of 1280 × 1024 pixels, at a distance of 54 cm from the eyes of the macaques, under conditions of head-fixation. The macaques performed a standard selective visual attention task described in more detail in.^23^ In brief, each trial was initiated when the macaques held a lever and foveated a white fixation spot (0.1°) displayed at the center of the screen on a gray background (1.41 cd/m^2^). Releasing the lever or breaking fixation for the duration of the trial led to the trial’s termination. After a pre-stimulus fixed delay period (duration of 614 and 674 ms, for macaques T and W, respectively), three peripheral colored square-wave gratings were presented at the same eccentricity and the same distance from each other, with one of the stimuli being centered on the RFs of neuronal populations in the V1 and V4 recording sites. We adjusted the diameter of the presented stimuli based on eccentricity and size of RFs, with stimulus diameters ranging from 2 to 4°. The color of each grating was pseudo-randomly permuted between recording sessions but remained fixed for the duration of each session. The gratings in the majority of recordings drifted perpendicular to the orientation of the grating, with the motion direction pseudo-randomly assigned on every trial. In 22 out of the total of 34 sessions, macaque W was shown stationary gratings. After a random delay (618–1131 ms for monkey T, 618–948 ms for monkey W; period duration chosen from a uniform distribution), a central colored cue appeared, matching the color of one of the peripheral gratings, thereby designating the target stimulus for the trial. The cue color was randomly assigned for each trial. At a random delay after the presentation of the cue, the luminance of one of the peripheral stimuli decreased (1162–2133 ms for macaque T, 1162–1822 ms for macaque W; period duration chosen from a uniform distribution). If the luminance change occurred on the target stimulus, the macaque was rewarded for subsequently releasing the lever. If, however, the luminance change occurred on one of the other two stimuli, the macaque was only rewarded after maintaining fixation and keeping hold of the lever until a luminance change occurred on the target stimulus, which corresponded to either the second or third luminance change event (each event following the previous luminance change after 792–1331 ms for monkey T and 792–1164 ms for monkeys W; period duration chosen from a uniform distribution).

##### Data acquisition: Macaques

Daily electrophysiological recordings from all cortical layers of visual areas V1 and V4 were performed using 16-contact linear electrode shanks (150 μm contact spacing, Atlas silicon probes), by inserting the shanks perpendicular to the cortex. Raw data were collected using an HS-36 Neuralynx pre-amplifier and a Neuralynx Digital Lynx amplifier with the Cheetah 5.6.3 data acquisition software interlinked with Remote Cortex 5.95. Data were sampled at 24 bit with a 32.7 kHz sampling rate and stored to a disc. We recorded eye position and pupil diameter at a rate of 220 Hz with a ViewPoint EyeTracker (Arrington Research).

##### Data acquisition: Mice

As mentioned above, analyses in mice were based on the Visual Coding Neuropixels dataset. This electrophysiology dataset comprises single unit spiking and LFP signals recorded simultaneously from 4 to 6 visual areas in 57 awake mice, under conditions of visual stimulation by various stimuli. In this study we focused on the interactions of the lateral geniculate nucleus (LGN), area V1, and area V2 (comprising the lateral (VISl), anterolateral (VISal), rostrolateral (VISrl), anteromedial (VISam), and posteromedial (VISpm) visual areas). Surgical procedures, visual stimulation protocols, recording equipment/techniques, signal preprocessing, and spike sorting have been extensively described in the technical white paper accompanying the dataset (https://portal.brain-map.org/explore/circuits/visual-coding-neuropixels), and will not be discussed here.

##### Analysis software

The analyses presented in this study were performed in MATLAB (The MathWorks) and used the FieldTrip analysis toolbox (https://www.fieldtriptoolbox.org).

#### Quantification And Statistical Analysis

##### Receptive field mapping: Macaques

The estimation of receptive fields (RFs) was based on the envelope of MUA (MUAe). This signal was extracted after low-pass filtering (5th order Butterworth filter with a corner frequency of 300Hz) the rectified 0.6–9 kHz filtered signal. RF mapping involved the presentation of 0.5–2° black squares on a 9 × 12 grid. An offline response map was computed for each channel via the reverse correlation of the MUAe signal to these stimuli. This map was then converted to z-scores, and RFs for each channel were defined as the region surrounding the peak activity that exceeded a *Z* score of 3. More detailed information about our approach in RF estimation can be found in.^77^

##### Preprocessing: Macaques

In macaques, we extracted local field potentials (LFPs) from the broadband signal by low-pass filtering (6th order Butterworth filter with a corner frequency of 500 Hz), high-pass filtering (3rd order Butterworth filter with a corner frequency of 2 Hz) and down-sampling to ~ 1.0173Hz. A Butterworth bandstop filter (50 Hz and harmonics ±0.2 Hz) was additionally used to remove powerline artifacts at 50 Hz and harmonics. Spike waveforms were extracted from the broad-band signal after taking the following steps: 1) The median-filtered signal (3ms window) was subtracted from the broadband signal. 2) This high-pass filtered signal was further band-pass filtered (second order Butterworth filter with corner frequencies of 20Hz and 8kHz). 3) The signal was further de-noised by subtracting the shank-wide median signal from each electrode in the corresponding electrode shank. 4) Individual spikes were detected as negative voltage crossings of a threshold of 5 absolute deviations. 5) Windows surrounding the local minima of these negative crossings were examined for the presence of early large positive peaks or double-negative peaks (window length of ±20 samples), and spikes with these characteristics were discarded. 6) Individual spike waveforms were defined as data points spanning –15 to 30 samples around the local minima that were not discarded.

##### Spike sorting: Macaques

We isolated single units (SUs) from macaque visual cortex in a semi-automated manner. Semi-automatic clustering was performed with the KlustaKwik software (version 1.7) on the following features: 1) The energy of the spike waveform and the energy of its first derivative. 2) The four principal components and the three Haar wavelet components explaining most of the variance of all detected spike waveforms. MClust (version 3.5) was used to manually assess the quality of isolation of each candidate cluster. Clusters were deemed to correspond to an isolated single unit if the following criteria were met: 1) The isolation distance (ID) of the cluster from the noise cluster^78^ exceeded a value of 15. 2) The L-Ratio (LR)^78^ of the cluster was lower than 0.2.

3) Less than 0.05% of inter-spike intervals were below 1 ms. The population of single units that were included in our analysis had a median ID of 26.1013 and median LR of 0.0406.

##### Waveform classification

The mean waveforms used in waveform classification were extracted by applying a median filter (window length of 3ms) on the broadband voltage trace, subtracting the median-filtered signal from the raw broadband signal, and computing the average across data segments of −20 to 96 samples around the timepoint corresponding to each SU spike. The mean waveform of each SU was then normalized by subtracting the median of the first and last 10 samples in the waveform, and subsequently dividing by the waveform’s energy. Triphasic waveforms characterized by a stronger early rather than late positive peak, and waveforms with a DC difference between their beginning and end segment were discarded. Two-dimensional t-Stochastic Neighbor Embedding (t-SNE; perplexity of 30) was then applied on the remaining waveforms. Lastly, fuzzy c-means clustering (fuzzifier of 2, euclidean distance metric, tolerance of 10^−10^) was performed on the t-SNE matrix, which resulted in two clearly separate waveform clusters, corresponding respectively to broad and narrow SU waveforms.

##### Spectral Analysis

Analyses involving LFPs, such as the computation of spectral power, and inter-areal LFP-LFP phase-locking, may be affected by the presence of electrode-headstage-related noise and the influence of the electrode reference ^79^. In this study, we addressed this issue by subtracting the average LFP signal across each recording electrode shank from the LFP signal of each corresponding electrode, in a trial-by-trial manner. After this preprocessing step, LFP-exclusive analyses were based on the multiplication of LFP data epochs of 0.5 s with seven distinct prolate Slepian tapers, and the subsequent application of the fast Fourier transform (FFT).^80^ Spectral power was computed by rectifying the resulting complex Fourier coefficients and raising them to the power of 2. The power spectra shown in Figure 3G were produced by multiplying power values, corresponding to different frequency bins, to the square of their respective frequencies. The phase-locking strength between LFPs in V1 and V4 was quantified with the Pairwise Phase Consistency (PPC) metric, which avoids pitfalls associated with standard measures such as the number of LFP epochs corresponding to each condition.^81^ In brief, PPC is computed in the following steps: 1) Phases of the V1 and V4 LFP signal are extracted from the complex Fourier coefficients corresponding to different trial epochs, and the V1-V4 relative phase is computed for each epoch. 2) The resulting complex vectors are normalized by their amplitude to produce unit vectors. 3) Conjugate multiplication is performed for each possible vector pair, and this product is then averaged and rectified. Note, that the peak frequency of stimulus-induced gamma depends on stimulus properties^82–84^ (Figure S1A) and individual subjects^85^ (Figure S1B). For this reason, we aligned mean power and V1-V4 LFP-LFP PPC spectra to the individual gamma peak of LFP-LFP phase locking separately for each session.

##### Filtering for gamma-band phase extraction and trial-by-trial estimation of phase-locking strength

In macaques, the estimation of the gamma-band LFP phases of spiking (Figure 1G), trial-by-trial spike-LFP phase-locking strength, trial-by-trial interareal LFP phase-locking strength, and the relative gamma band phases of V1 and V4 LFPs (the latter four used in decoding analyses; Figures 4B and 4C) was based on using a two-way, bandpass Butterworth filter with an order of 12. As noted above, the two macaques used in this study exhibited different gamma-band peak frequencies, thus we used different corner frequencies for each macaque (40–90 for monkey T, 25–55 for monkey W). Next, we performed the Hilbert transform on the filtered data. Note that filtering and the Hilbert transform were applied on the complete trial before selecting relevant trial epochs for further analysis. The results shown in Figure 1G were produced by collecting the time points of the occurrence of each SU spike and the computation of the mean angle across all concurrent V1 Hilbert-transformed LFP datapoints. See the ‘decoding analyses‘ section below for further information about the analyses of filtered signals in decoding analyses.

In mice, LFP phases of SU spiking were extracted from the complex Fourier coefficients corresponding to the gamma-band peak frequency in the SU-LFP PPC spectrum.

##### Quantification of Spike-LFP phase-locking: Macaques

Spike-LFP phase locking in macaques was assessed for the period between post-stimulus onset and target change for Figures 1D–1F, 2B, 2C, 5B, S2A, and S2C (period between cue onset and target change for Figures 3E, 3H, S2B, S4 and S6). In these analyses, we used bipolar derivatives of the LFP, in order to mitigate the effect of $\frac{1}{{\text{f}}^{\text{n}}}$ noise and lower frequency oscillations on the estimation of gamma-band spike-LFP phase-locking strength. The strength of spike-LFP phase locking was assessed with PPC, and in particular the PPC1 measure,^81^ which removes bias associated with differences in spiking rates. In short, the LFP-phase of SU spiking for each frequency f, was ascertained by collecting LFP data segments of a duration of $\frac{9}{\text{f}}$s centered around each spike. We, then, multiplied these LFP segments with a Hann taper of a corresponding length, and performed an FFT on the resulting product. Only SUs with ≥ 200 spikes per condition were included in our analyses. This resulted in the inclusion of a different number of cells in different analyses, e.g., spectra corresponding to NW cells in Figures 2B and 3E involved different numbers of cells. We performed additional analyses in order to control for the effect of spiking rate on spike-LFP phase locking, when comparing different cell types and attention conditions (Figures 2C and S4, respectively). For the comparison between the spike-LFP phase-locking strength of BW and NW cells, we separately weighted the PPC spectrum of each cell by the ratio of its spike count and the mean spike count of each cell in the same cell class. For the comparison between attention conditions, the PPC spectrum of each cell per condition was weighted by the ratio of its spike count for the respective condition and the mean total spike count of each cell included in the analysis.

##### Quantification of Spike-LFP phase-locking: Mice

In mice, spike-LFP phase locking for the luminance condition was assessed as in.^28^ Briefly, data periods during the presentation of a gray screen were divided into 1s pseudo-trials. Phase locking in the contrast condition was estimated for the period between the onset and offset of each grating stimulus. Note, that the LGN lacks the ordered columnar structure found in cortical areas and, thus, does not produce an LFP signal. Therefore, in order to examine population-wide oscillatory activity in the LGN, we estimated a surrogate LFP (sLFP) signal derived from population spiking.^28^ This was done by summing the spikes of all individual isolated units in the LGN, and filtering this signal between 1 and 100Hz. We exclusively analyzed sessions where at least 10 single units were recorded in LGN. As in the macaque, spike-LFP phase locking was quantified by the PPC1 metric. This was done after segmenting the LFP in data epochs of 250ms, multiplying these segments with a Hann taper and performing the Fourier transform.

##### Drivenness, attentional effect in spiking, and stimulus selectivity indices

SU peri-event time histograms (PETHs) for different trial epochs (Figures 3A, 3B, 3F, and 6A) were computed by taking the following steps: 1) We counted the total number of spikes in bins of 1ms for the full trial. 2) The resulting time series were convolved with a Gaussian kernel with a length of 50ms, and a standard deviation of 8ms. 3) We computed trial-wise z-scores of the smoothed time series. 4) Relevant trial epochs were collected and averaged across trials.

SU drivenness by the stimulus (Figure S2A) was estimated by using the following equation:

$$\text{dr}=\frac{{\text{FR}}_{\text{stim }}-{\text{FR}}_{\text{base }}}{{\text{FR}}_{\text{stim }}+{\text{FR}}_{\text{base }}}$$

where FR_stim_ and FR_base_ respectively designate the mean spike-count in the period between 0.05 and 0.25s after stimulus onset and a period spanning 0.2s before stimulus onset. The attentional modulation index (AMI) was computed based on the following equation:

$$\text{AMI}=\frac{{\text{FR}}_{\text{tow }}-{\text{FR}}_{\text{away }}}{{\text{FR}}_{\text{tow }}+{\text{FR}}_{\text{away }}}$$

where FR_tow_ and FR_away_ designate the mean spike-count in the 1s period before the first stimulus change across trials (Figures 3C, 5A, S2B, and S2C; 0.15s–0.25s and 0.35–0.45s after cue onset in Figure 6B), respectively for the attend-toward and attend-away conditions. The spiking rates shown in Figure 3C and used in decoding analyses were computed for the same time period. Indices quantifying the selectivity of SUs, recorded in mice, to grating properties (orientation, direction, and temporal frequency) were included in the Visual Coding Neuropixels dataset provided by the Allen Institute for Brain Science.

##### Decoding analyses: Macaques

Decoding of the macaque’s attentional state was focused on the time period spanning 1s before the onset of the first luminance change event. In our decoding analyses, we compared the decoding accuracy of 5 physiological measures: 1) SU spiking rates. 2) The strength of V1- V4 gamma-band LFP-LFP phase-locking. 3) The relative phase of V1 and V4 gamma-band LFPs. 4) The strength of the gamma-band phase-locking of V4 SU spiking to V1 LFPs. 5) The phase of V1 gamma-band LFPs in which V4 SU spiking occurred. Note that decoding analyses involving LFPs were performed on filtered and Hilbert-transformed signals, as described in section ‘filtering for gamma-band phase extraction and trial-by-trial estimation of phase-locking strength‘. The strength of phase-locking between LFPs in each trial was estimated by computing the Phase Locking Value (PLV) between the gamma-band complex time series corresponding to each 1s epoch for all possible V1-V4 LFP pairs. The trial-by-trial relative phase between each V1 and V4 LFP pair was computed after performing a timepoint-by-timepoint conjugate multiplication between the two complex vectors corresponding to each LFP pair, averaging the values in the resulting complex vector, and estimating its angle. The strength of phase-locking between V4 spiking and each gamma-band LFP in V1 was assessed in each trial by collecting the complex coefficients of the LFP corresponding to each V4 spike in the trial and computing PPC between them. Spikes in this analysis were pooled across simultaneously recorded SUs. Lastly, the V1 gamma-band phase of V4 SU spiking was estimated in each trial by computing the mean angle of complex LFP coefficients corresponding to each V4 SU spike for every SU-LFP pair.

Our decoding analyses used a maximum-likelihood (ML) estimation algorithm described in more detail in.^86^ In short, the ML algorithm depends on Bayes’ rule:

(Equation 1) $$\text{P}(\alpha |{\text{A}}_{\text{pop}})=\frac{\text{P}({\text{A}}_{\text{pop}}|\alpha )\text{P}(\alpha )}{\text{P}({\text{A}}_{\text{pop}})}$$

where P(*α*), is the prior, which designates the prior probability of the trial corresponding to the attend-toward condition; P(*α*|A_pop_) is the posterior, which designates the posterior probability of the trial’s neural activity A_pop_ being observed because the trial belongs to the attend-toward or the attend-away condition; P(A_pop_|*α*), is the likelihood, which designates the probability that the attentional condition will result in neural activity A_pop_; and P(A_pop_) is the model evidence, which is the probability of observing neural activity pattern A_pop_. For our analyses, we used only flat priors.

As mentioned above, we performed decoding analyses by either using angular quantities (relative phase of V1 and V4 LFPs or the LFP phase of spiking), the spiking rate, or other non-angular quantities (strength of phase-locking between V1 and V4 LFPs, or strength of phase-locking between spiking and LFPs). We analyzed angular quantities for any neural signal i by approximating the likelihood distribution for the allocation of attention to either the stimulus driving V1 and V4 neural activity or one of the other two stimuli by a von Mises distribution with a location *μ* and concentration *κ*. For spiking rates and non-angular quantities the likelihood was approximated, respectively, by a Poisson distribution with an expected occurrence rate *λ* and a Gaussian distribution with a mean *μ* and standard deviation *σ*. The decoder was trained in a leave-one-trial-out jackknife fashion.

For decoding based on population activity, we focused on the spiking rates of simultaneously recorded SUs, all possible V1 and V4 LFP pairs, and the phases of spiking of simultaneously recorded SUs in V1 gamma-band LFPs. Decoding accuracy was estimated as a function of the number of simultaneously recorded SUs by random subsampling as in.^86^ The posterior probability distribution for the attend-toward condition corresponding to a population of n signals can be estimated by computing the product over the posterior probabilities for all n signals:

(Equation 2) $$P(a|A_{pop})\propto \prod_{i=1}^{n}P{\left(a|A\right)}_{i}$$

The decoded attentional condition is then defined as the condition with the largest population-wide posterior probability.

##### Trial-by-trial decoding error

The potential co-fluctuation in decoding performance between SU spiking and the phase-locking strength of LFPs, or their relative phase, was assessed via the estimation of the trial-wise decoding error of these physiological quantities (Figure S5). More specifically, we first computed the decoding error (whether the decoded attention condition matched the actual attention condition) related to the spike-count of each SU, separately for each trial. Similarly, we computed the population-wide decoding error of the PLV between gamma-band LFPs in V1 and V4, or the relative phases of these LFP pairs. We then computed Spearman’s rho between the decoding error of the spike-count of each SU and the population-wide decoding error of LFP-related measures, across all of the trials in which a given SU was detected.

##### Assignment of cortical layers

The assignment of neuronal activity to either the superficial, granular or deep laminar compartment was primarily based on the extraction of the current source density (CSD) signal from the stimulus-evoked LFP signal. In mice, CSD was extracted under conditions of whole screen flash stimulation, whereas in macaques this was done during the period after the onset of the peripheral grating stimuli used in the attention task. Stimulus-evoked CSD was computed by taking the second discrete spatial derivative of the LFP across different electrodes in each electrode shank.^87^ LFPs from electrode contacts with a relatively low signal-to-noise ratio were discarded and exchanged with a signal derived from a linear interpolation between the contacts’ neighboring electrodes. Further, in macaques, recording sessions that displayed a substantial drift of RF location with cortical depth were excluded from our laminar analyses.

In addition to the identification of sinks and sources in the stimulus-evoked CSD, different cortical laminar compartments were identified in the macaque through the inspection of the latency of stimulus-evoked multi-unit activity at different cortical depths. Peri-stimulus time histograms in this analysis included all detected negative spikes from each electrode contact and were computed similarly to other PETHs shown in our study, but with a Gaussian kernel with a length of 25 ms and a standard deviation of 4 ms. Finally, we computed the logarithm of the ratio between the total number of positive spikes and the total number of negative spikes, detected in each electrode contact. Contacts with a log-ratio close to zero were deemed to lie outside of the brain, whereas contacts with negative log-ratio were assigned to the gray matter, and contacts with a positive log-ratio were presumably located in the white matter.

##### Multi-linear regression

We investigated the relationship between the attentional modulation of SU spiking and other physiological properties of V4 SUs (Table S1). This relationship was quantified in the form of a multi-linear model that aimed to predict each cell’s AMI, by using their baseline firing-rate, their laminar location, and the interaction of baseline firing-rate and laminar location as predictors. The model was fit to the data with a least-squares regression process implemented in MATLAB in the function *regstats*.

##### Analysis of latency in attention effects

The latency of attentional effects on the firing-rate of SUs in different laminar compartments/areas was examined in two ways: (1) We computed the difference between SU peri-cue-time-histograms (−0.1-+0.5s relative to cue onset) corresponding to the two attention conditions (Figures S7A and S7B). This was done separately for, both, non-normalized and z-scored histograms. Note that, difference-time-courses may be substantially noisier than the original PETHs. For this reason, PETHs in this analysis underwent smoothing with a Gaussian kernel of a length of 80 samples and a standard deviation of 13 samples. The presence of statistically significant differences between these difference-time-courses at particular time-bins was assessed in the same way as for other PETHs shown in this study (see STAR Methods: statistical testing). (2) The abovementioned difference-time-courses were further used in a lagged cross-correlation analysis implemented with the unbiased version of the MATLAB function *xcorr* (Figures S7C and S7D). For this analysis we considered the period between 0.05 and 0.5s after the onset of the cue. The resulting cross-correlation functions were subsequently appraised for the presence of significant asymmetries present close to the center of each function. We defined asymmetry as the difference between the integrals of the cross-correlation function at the −0.2-0s and the 0-0.2s intervals. Statistical significance in a given asymmetry was determined via a randomization test, where cells comprising each population were pseudorandomly reassigned to each population in an iterative manner (1000 iterations). Both analyses described above excluded cells with firing rates that were either not significantly modulated by attention, or were negatively modulated by attention.

For the comparison of latencies of attentional effects in V1-V4 phase-locking and SU V4 spiking rates (Figure S3) we took the following approach: (1) For phase-locking we used Hann windows with lengths of ±2.5 cycles per frequency which were slid over the available data in steps of 1 ms. The resulting time-frequency PPC representations were aligned to each session’s gamma peak frequency (Figure S3A; see Methods: Spectral Analysis for further details). For Figure S3B we computed the mean across frequencies between −10 and +10 Hz around the gamma peak-frequency of each session. (2) V4 PETHs (Figure S3C) underwent smoothing with a Hann kernel of a length of 125 ms. This length was chosen to correspond to 5 cycles of the mean gamma peak-frequency across sessions −10Hz (i.e., 40 Hz), and therefore to enable a direct comparison between the PETHs and the gamma-band PPC time-series.

##### Computation of noise-correlation and Granger-causality

Spike-count correlations and Granger-causality were estimated on multi-unit activity, that is, all negative voltage-crossings in the high-pass filtered signal (see Section preprocessing: macaques for more details). This was done in order to maximize the signal-to-noise ratio in our analyses. The magnitude of spike-count correlations across trials (i.e., noise-correlations) was assessed, both, in a time-resolved manner (Figures S8A and S9A) and in fixed time-windows (Figures S8B and S9B). In our time-resolved analyses we counted the number of detected MU spikes in a window of ±40 ms, slid every 10 ms. Next we computed Pearson’s correlation coefficient between the resulting time-series for any given channel pair. Note that task-independent, slow dynamics in spiking can influence the magnitude of a correlation, if the latter is computed for all trials in a given session. In order to mitigate such session-wide effects we computed Pearson’s r separately for chunks of ten adjacent trials, and subsequently averaged the resulting r across these trial-chunks. Similarly, analyses in fixed time-windows (length of 0.5s) were also based in the computation of Pearson’s r across ten-trial chunks and subsequent averaging.

Inter-areal and inter-compartmental directed influences in spiking activity (Figure S10; Table S2) were determined through the use of multivariate Granger-causality, and, in particular, its non-parametric version.^88^ More specifically, we collected MU time-series in fixed windows of 0.5s, and computed their Fourier spectra for frequencies between 4 and 200 Hz in bins of 2 Hz, after multiplying the time-series with a Hann taper. The resulting Fourier spectra were then subjected to the non-parametric spectral matrix factorization process, as it is implemented in the Fieldtrip toolbox. It should be noted that Granger-causal directionality may be detected, in the absence of genuine directional influences, if one of the areas under consideration exhibits relatively higher levels of noise in its activity.^89^ We assessed whether such an artifactual influence is present in our data by reversing the MU time-courses used in our analyses and recomputing the multivariate Granger-causality metric. In cases where genuine Granger-causal directionality is indeed present, this time-reversal leads to a significant reversal of the detected Granger-causal directionality. We further note that the estimation of the magnitude of Granger-causal influences may be influenced by the total number of trials used for a given analysis. We controlled for such an influence when comparing attention conditions by equalizing the number of trials corresponding to each condition.

##### Testing for optogenetic response: Mice

Optogenetic tagging experiments were performed on mutant mice expressing Pvalb-IRES-Cre and Sst-IRES-Cre. The optogenetic stimulation consisted of 10 ms pulses (for more details, see https://portal.brain-map.org/explore/circuits/visual-coding-neuropixels). Cre-expressing cells were identified using the ZETA-test,^90^ a recently developed parameter-free statistical test to determine whether neurons exhibit a time-locked modulation of firing rates by a specific event. First, the ZETA test was used to test which neurons showed significantly modulated spiking activity (*p* < 0.05) within a 0.5-s window following the onset of optogenetic stimulation. Next we calculated the instantaneous peak- and trough-latencies of all significantly modulated cells. Cells were classified as optogenetically tagged if their peak latencies occurred within the 10 ms of optogenetic stimulation. To avoid misclassification due to laser artifacts, neurons with a peak earlier than 1 ms after the onset of the optogenetic pulse were discarded.

##### State detection: Mice

The strength of luminance and contrast gamma in the mouse was positively modulated by arousal and locomotion, respectively (Figures S11A, S11B, S12B–S12G, and S13), therefore this analysis was focused on periods of high locomotion, in order to optimize the signal-to-noise ratio of gamma LFPs. We assessed the arousal level and running speed of mice by examining, respectively, the pupil diameter signal and speed signal accompanying the Visual Coding Neuropixels dataset, used in our study. For the luminance condition, we detected periods of high and low arousal by normalizing the pupil diameter signal by its maximum value, separately for each recording session, and classified periods in which this signal had values between 0.65 and 0.95 as periods of high arousal (values between 0.3 and 0.55 corresponded to periods of putative low arousal). For the contrast condition, states of high and low locomotion were defined as periods in which the mouse had a running speed > 5 cm/s and < 1 cm/s, respectively.

##### Statistical testing

The statistical tests are reported in the respective figure legends and the results text. The degrees of freedom (N) are specified in the figure legends and correspond to either the number of cells or sessions. The definitions of center and precision measures are also mentioned in the figure legends. The statistical tests that we used were nonparametric and therefore did not rely on specific assumptions about the distribution of the data. Unless otherwise stated, statistical comparisons in the study were non-parametric, two-sided, and based on 1000 randomizations.^91^ Randomization between means of quantities (spectra, spiking rate, decoding accuracy, AMI) measured across different cell populations/types (Figures 1D, 1E, 2B, 2C, 4A, 5A, 5B, 6B, 7B, 7D–7F, S11A, S11B, S12B–S12G, S13, and S14) and trial-epochs (Figure 1C) involved randomly exchanging the quantities under comparison between populations or epochs, while keeping the original number of values per population/epoch constant. In the case of spectra, statistical significance was achieved for frequency bins where observed differences between the mean spectra of each population were larger or smaller than the 97.5th percentile of the maximal values or the 2.5th percentile of the minimal values, respectively, across all randomized difference-spectra. This approach corrects for the false discovery rate associated with multiple comparisons. In the case of singular values per cell (e.g., spiking rate) we computed *p*-values by taking the following steps: 1) We computed the ratio of the mean difference between populations and the standard deviation across all randomized differences. 2) We rectified this ratio and computed its cumulative density function (CDF) value. 3) We subtracted this value from 1 and divided the difference by 2. The statistical assessment of correlations (Figures S2 and S6) involved randomly shuffling the order of the PPC values corresponding to each cell and computing Spearman’s rho 1000 times. Here, *p*-values were computed in a similar manner as described above, with the main difference being that we compared the empirical mean correlation to a distribution of randomized correlations. Statistical comparisons between attention conditions (Figures 3A, 3B, 3D–3H, 6A, and S4 were also done similarly to what was described above, with the only difference being that randomizations were based on the random switching of attentional condition labels.

## Supplementary Material

Supplemental Materials

## Highlights

- Macaque V1-V4 recordings reveal laminar and cell-type specific effects of attention
- Layers II/III neurons in V4 show strongest and earliest attentional rate modulation
- Layer IV interneurons in V4 but not excitatory neurons phase-lock to V1 gamma rhythm
- Mice and monkeys show similar laminar and cell-type specific synchronization patterns

## In brief

Spyropoulos et al. show that attention modulates V1-V4 firing rates and gamma phase-locking via distinct feedforward and feedback pathways. Firing-rate modulation is strongest and earliest in Layers II/III of V4, consistent with feedback. Gamma phase-locking to feedforward V1 gamma is confined to fast-spiking interneurons in Layer IV of V4.
