# Supplementary material for: Distinct feedforward and feedback pathways for cell-type specific attention effects
Source: Neuron. Author manuscript; Available in PMC 2024 Nov 26. (PMC7616856; doi:10.1016/j.neuron.2024.04.020)
Supplement: Supplemental Materials [file EMS200280-supplement-Supplemental_Materials.zip › 1-s2.0-S0896627324002812-mmc1.pdf]

Neuron, Volume *112*

Supplemental information

Distinct feedforward and feedback pathways

for cell-type specific attention effects

Georgios Spyropoulos, Marius Schneider, Jochem van Kempen, Marc Alwin Gieselmann, Alexander Thiele, and Martin Vinck

Supplementary Information for: Distinct feedforward and feedback pathways for cell-type specific attention effects

Georgios Spyropoulos[a,d], Marius Schneider[a,b,d], Jochem van Kempen[c], Marc Alwin Gieselmann[c], Alexander Thiele[c], Martin Vinck[a,b,e]

[a] *Ernst Strüngmann Institute (ESI) for Neuroscience in Cooperation with Max Planck Society, 60528 Frankfurt am Main, Germany*
[b] *Donders Centre for Neuroscience, Department of Neuroinformatics, Radboud University Nijmegen, 6525 Nijmegen, Netherlands*
[c] *Biosciences Institute, Newcastle University, Newcastle upon Tyne NE1 7RU, UK*
[d] *These authors contributed equally to this work*
[e] *Correspondence to martin.vinck@esi-frankfurt.de*

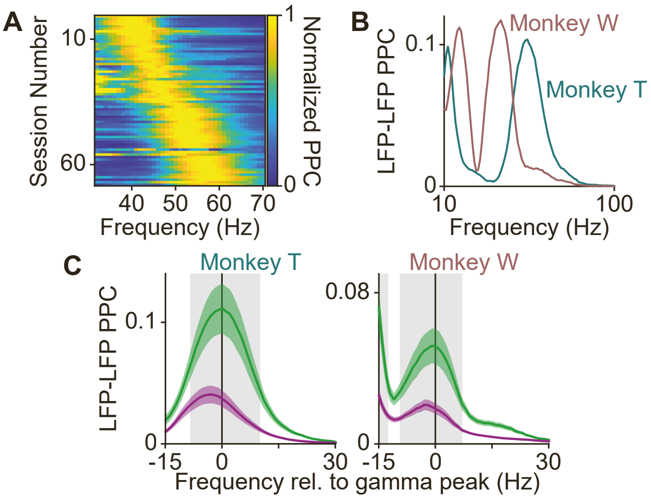

Fig. S1: Variability in the gamma-range peak-frequency of LFP-LFP phase-locking in macaques, related to Figures 1 and 3. **(A)** Normalized LFP-LFP PPC between V1 and V4 across all inter-areal channel pairs, for all sessions (N= 68), sorted for gamma-range peak-frequency. **(B)** Example sessions demonstrating the difference in gamma-range peak-frequency in the two monkeys. **(C)** Same as Figure 3D but plotted separately for the two monkeys (N= 34 sessions for each monkey).

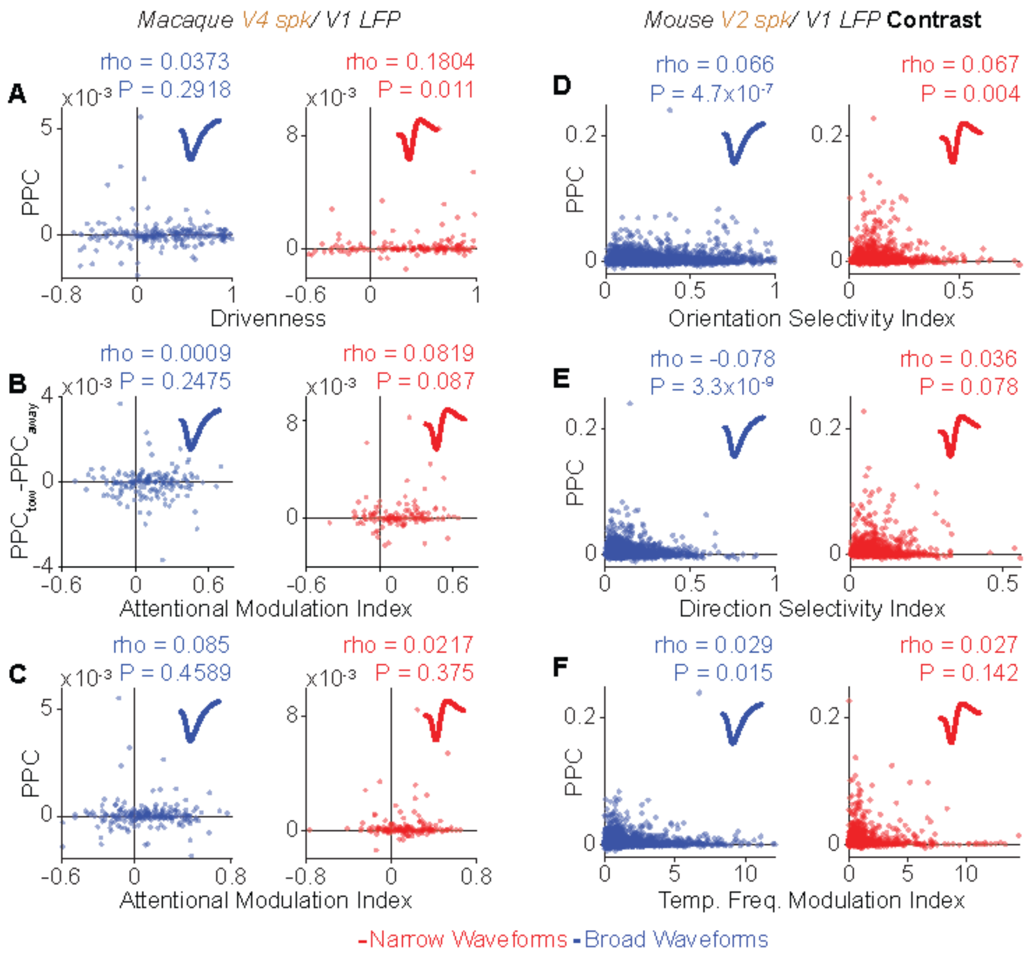

Fig. S2: Effects of cell-drivenness and the attentional modulation of spiking on the gamma-rhythmic phase locking between downstream spiking and upstream LFPs for different cell types in macaques and mice, related to Figures 2, 3 and 7. (A) Relationship between cell-drivenness and gamma-band PPC of spiking to V1 LFPs for V4 BW and V4 NW cells (BW: N= 216; NW: N= 152). **(B)** Same as A, but for the attentional modulation index of firing rates and the attentional difference of the gamma-band PPC of spiking and V1 LFPs (BW: N= 162; NW: N= 134). **(C)** Same as A, but for the AMI of V4 spiking and gamma-band PPC of spiking to V1 LFPs (BW: N= 216; NW: N= 152). **(D)** Same as A, but for the orientation selectivity index of spiking and gamma-band PPC of spiking to V1 LFPs for V2 BW and V2 NW cells, in the mouse. **(E)** Same as C, but for the direction selectivity index of spiking and gamma-band PPC of V2 spiking to V1 LFPs. **(F)** Same as C, but for the depth of modulation of spiking by the temporal frequency of the grating stimuli and gamma-band PPC of V2 spiking. **(A-F)** Correlations were assessed with Spearman's rank correlation coefficients (rho). Statistical significance was assessed with a randomization test across cells. **(D-F)** BW: N= 5477; NW: N= 1585.

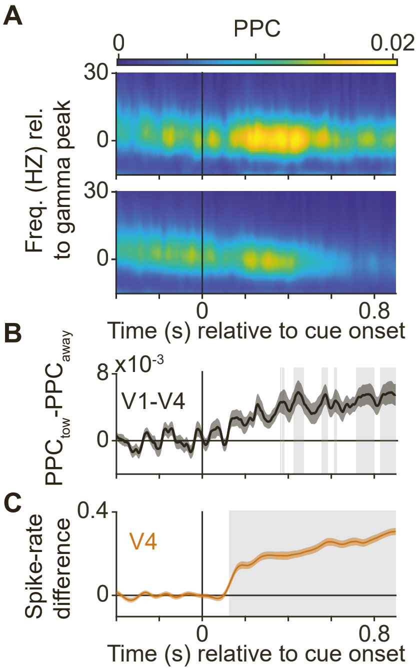

Fig. S3: Attention-related increases in SU firing rate precede increases in phase-locking between LFPs in V1 and V4, related to Figure 6. **(A)** Mean PPC between V1 and V4 LFPs as a function of time relative to the onset of the attentional cue and frequency relative to the gamma peak, illustrated separately for the attend-toward (top) and attend-away (below) conditions. **(B)** Average time-course of the difference of gamma-band V1-V4 PPC between the two attention conditions. Significance was assessed with a randomization test across sessions (N= 68; FDR correction for multiple comparisons with a significance threshold of $P < 0.05$). Confidence intervals designate SEM across sessions. Note that the first significant bin appears at a latency of +0.363s. **(C)** Average time-course of the difference of V4 spike rates between the two attention conditions. Significance was assessed with a randomization test across significantly and positively modulated cells (N= 215; FDR correction for multiple comparisons with a significance threshold of $P < 0.05$). Confidence intervals designate SEM across cells. Note that the first significant bin appears at a latency of +0.124s.

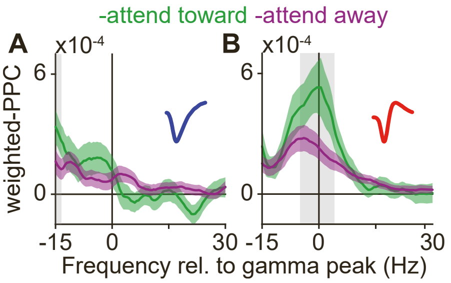

Fig. S4: Cell-type specific effects of attention on gamma-rhythmic synchronization persist after controlling for SNR, related to Figure 3. (A,B) Same as Figure 3E, but after weighting the PPC spectrum corresponding to each cell by the cell's number of spikes.

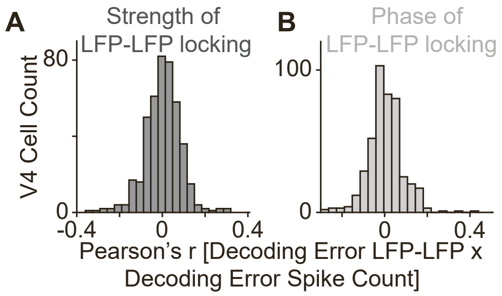

Fig. S5: Relationship in trial-by-trial decoding performance between SU spike-counts and inter-areal LFP synchronization, related to Figure 4. (A) Distribution of correlation coefficients (Pearson's r) quantifying the trial-by-trial relationship in the decoding error of SU spike-counts and PLVs of V1 and V4 LFPs. The mean of the distribution is statistically indistinguishable from zero (mean= 9.55×10^{-4}, P= 0.4574). **(B)** Same as A but for SU spike-counts and the relative phase of V1 and V4 LFPs (mean= 0.058, P= 0.1809). **(A,B)** Wilcoxon's signed-rank test across cells, N= 420.

A BW

$V4_{SUP}$-$V4_{GRA}$	beta	t-statistic	P-value
**Basesine FR**	-0.04064	-1.06795	0.288369
**Compartment**	-0.08586	-2.72179	**0.007781**
**Interaction**	0.0119	1.06339	0.290419

B NW

$V4_{SUP}$-$V4_{GRA}$	beta	t-statistic	P-value
**Baseline FR**	0.06444	1.396199	0.167886
**Compartment**	0.00554	0.12244	0.902966
**Interaction**	-0.0283	-1.93637	0.057617

Table 1: The relationship between the attentional modulation of SU spiking, their baseline firing-rate, and their laminar location, related to Figure 5. (A) Results of multi-linear least-squares regression of the AMI of BW SU spiking, using SU baseline firing-rate, the laminar location of SUs, and the interaction of the latter two variables as predictors. The different columns display the beta coefficients of the linear model (left), the respective t-statistics (middle), and respective P-values (right). P-values lower than 0.05 are displayed in a bold font. N= 52 for the superficial compartment, and N= 42 for the granular compartment. **(B)** Same as A, but for NW cells. $N = 34$ for the superficial compartment, and N= 30 for the granular compartment.

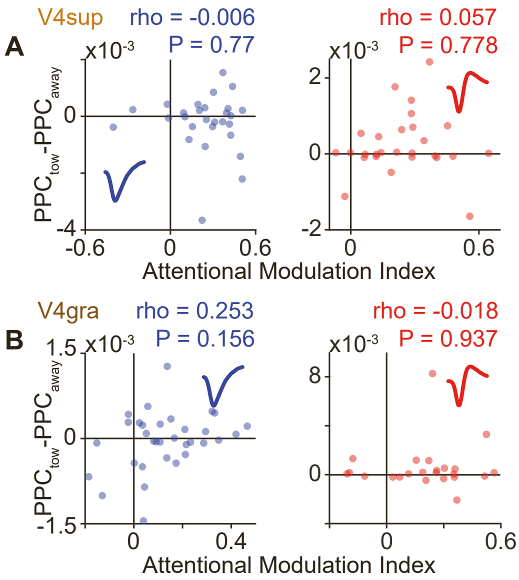

Fig. S6: Relationship between the attentional modulation of spiking and the attentional modulation of gamma-rhythmic phase-locking between downstream spiking and upstream LFPs for different laminar compartments in macaque V4, related to Figure 5. (A) Correlation between the attentional modulation index of firing rates and the attentional difference of the gamma-band PPC of spiking and V1 LFPs, for BW cells (left; N= 28) and NW cells (right; N= 27) in the superficial compartment of V4. **(B)** Same as A, but for BW cells (left; N= 33) and NW cells (right; N= 23) in the granular compartment of V4. **(A-B)** Correlations were assessed as in Figure S2.

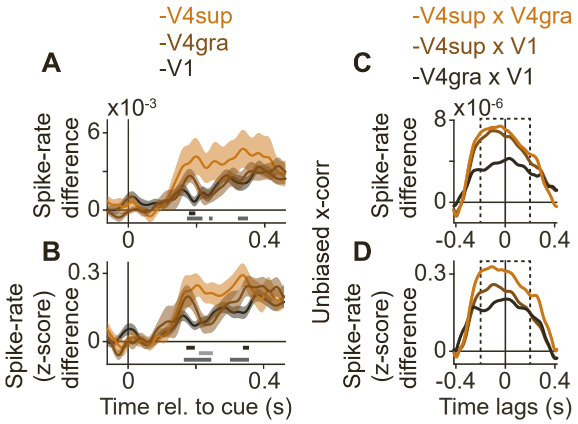

Fig. S7: Attention-related elevation in the firing rate of significantly and positively modulated SUs appears earliest in the superficial layers of V4, related to Figure 6. **(A)** Time-course of the difference in firing rates between the two attention-conditions, for SUs that display a positive and significant modulation in their firing rates. We considered SUs in the superficial layers of V4 (orange, N= 24), in the input layer of V4 (brown, N= 25), and area V1 (black, N= 53). **(B)** Same as B but for the difference between the z-score of firing rates. **(C)** Cross-correlation of firing-rate differences between SUs in the superficial and the granular compartment of V4 (orange), the superficial compartment of V4 and V1 (brown), and the granular compartment of V4 and V1 (black). Cross-correlations were computed for the period between 0.05 and 0.5s after the onset of the cue, and were based on the average time courses shown in (A) and (B). The asymmetry in cross-correlations, which indicates the lead/lag relationship of time-courses, was assessed for the window of ± 0.2s around the center of each cross-correlation function. In this analysis, the cross-correlation between the granular compartment of V4 and V1 shows no significant asymmetry (P= 0.2062), whereas the superficial compartment of V4 leads, both, V1 and the granular compartment of V4 (P= 0.0399 and P= 0.0174, respectively). **(D)** Same as B but for the difference between the z-score of firing rates. Here, the cross-correlations between the granular compartment of V4 and, both, V1 and the superficial compartment of V4 show no significant asymmetry (P= 0.2326 and P= 0.066, respectively), whereas the superficial compartment of V4 leads V1 (P= 0.0099). **(A,B)** Orange, brown, and black horizontal bars designate time-bins, that exhibit a statistically significant difference between SUs in the superficial and granular compartment, the superficial compartment and V1, and the granular compartment and V1 compartment, respectively (randomization test between SUs, FDR correction for multiple comparisons with a threshold of P< 0.05). **(C,D)** Statistical significance was determined with a randomization test between cells.

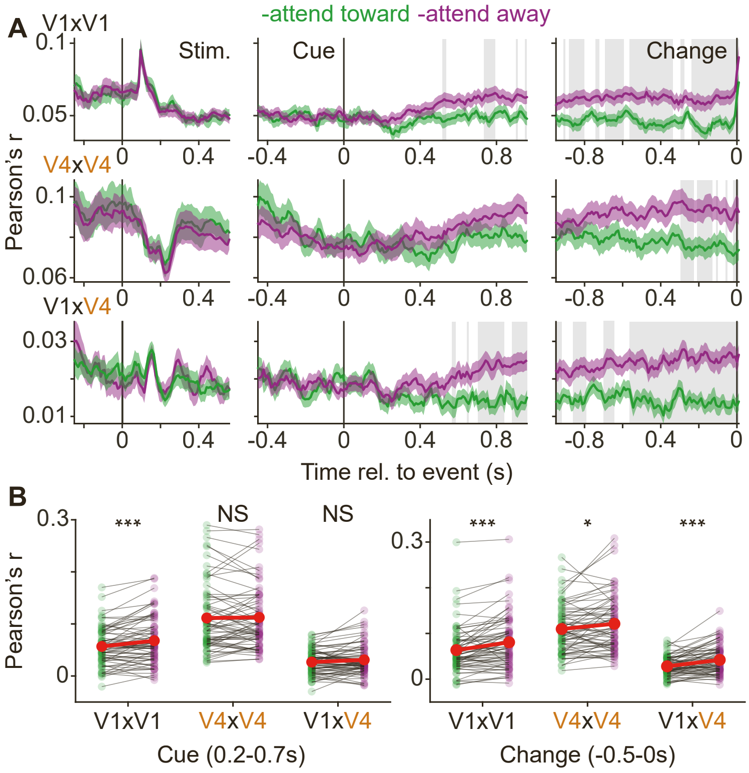

Fig. S8: Attention decreases the strength of intra- and inter-areal noise correlations, related to Figures 3 and 6. (A) Event-triggered time-course of spike-count correlations (Pearson's r) of MUs across trials, for the two attention conditions. The panels depict correlations, respectively, between MUs in V1 (top), V4 (middle), and V1-V4 MU-pairs (bottom). Confidence intervals designate SEM across sessions (N= 68). Gray rectangles designate significantly different time bins (randomization test between sessions, FDR correction for multiple comparisons with a threshold of P< 0.05). **(B)** Spike-count correlations (Pearson's r) of MUs across trials, for the period between 0.2 and 0.7s after cue-onset (left), and the period of 0.5s before the first stimulus change (right). *P< 0.05, ***P< 0.001; Wilcoxon's signed-rank test across sessions (N= 68).

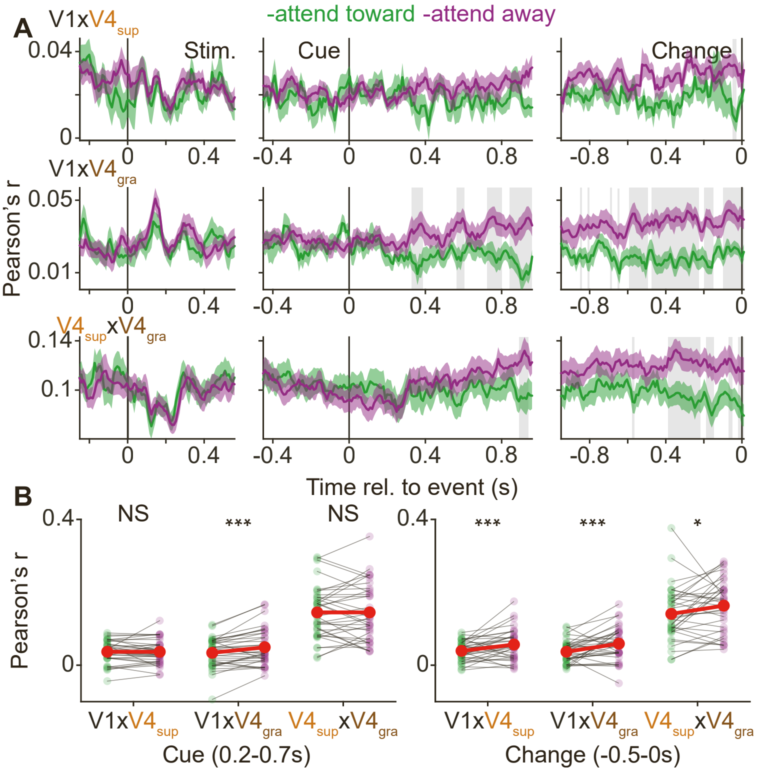

Fig. S9: Attention decreases the strength of noise correlations between V1 and different laminar compartments in V4, related to Figures 3, 5 and 6. (A) Same as Figure S8A but for MU-pairs in V1 and superficial layers of V4 (top), V1 and the input layer in V4 (middle), and MUs in superficial layers and the input layer of V4 (bottom) (N= 35). **(B)** Same as Figure S8B but for distinct laminar compartments as described in A (N= 35).

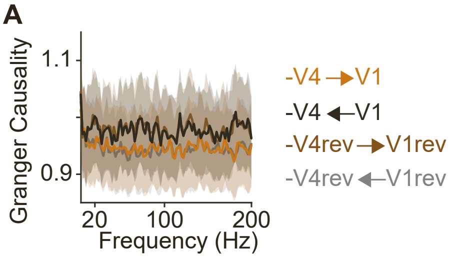

Fig. S10: Granger Causality between spiking activity in V1 and V4 displays no spectral peaks, related to Figure 1. (A) Multi-variate Granger causality spectra between multi-unit activity (MUA) in V1 and V4. Note the absence of spectral peaks. Orange, black, brown, and grey spectra depict the respective Granger-causal influence from V4 to V1, V1 to V4, V4 to V1 after time-reversing MUA time-series, and V1 to V4 after time-reversing MUA time-series. Confidence intervals designate SEM across sessions (N= 68).

A

**Attend-toward**				
Cue Onset +0.15s				
**Directionality**	V4→V1	V4←V1	V4→V1rev	V4←V1rev
**Granger Causality**	0.9342	**0.9786**	**0.9792**	0.9332
**P-value**		**4.74x10^{-4}**		**0.0023**

**Attend-away**				
Cue Onset +0.15s				
**Directionality**	V4→V1	V4←V1	V4→V1rev	V4←V1rev
**Granger Causality**	0.9473	**0.9776**	**0.9796**	0.9462
**P-value**		**6.8x10^{-4}**		**1.5924x10^{-4}**

B

**Attend-toward**				
1st Change -0.5s				
**Directionality**	V4→V1	V4←V1	V4→V1rev	V4←V1rev
**Granger Causality**	0.9587	0.954	0.9578	0.9545
**P-value**		0.612		0.4377

**Attend-away**				
1st Change -0.5s				
**Directionality**	V4→V1	V4←V1	V4→V1rev	V4←V1rev
**Granger Causality**	0.9686	**0.9951**	**0.9916**	0.9721
**P-value**		**0.006**		**0.034**

Table 2: Granger-causal influence from V1 to V4 decreases with attention, related to Figure 6. (A) Multivariate Granger-causality (GC) between MUs in V1 and V4 during the time-window between 0.15 to 0.65s after cue onset. GC was computed for, both, the original and time-reversed MU time-series, and is displayed separately for the attend-toward (left) and the attend-away condition (right). Statistical significance in directionality was assessed for, both, the original and time-reversed time-series with Wilcoxon's signed-rank test across sessions (N= 68). The reported P-values in each panel assess directionality, respectively, for the original MU time series (left) and the time-reversed control time-series (right). **(B)** Same as A, but for the time-window of 0.5s before the first stimulus change.

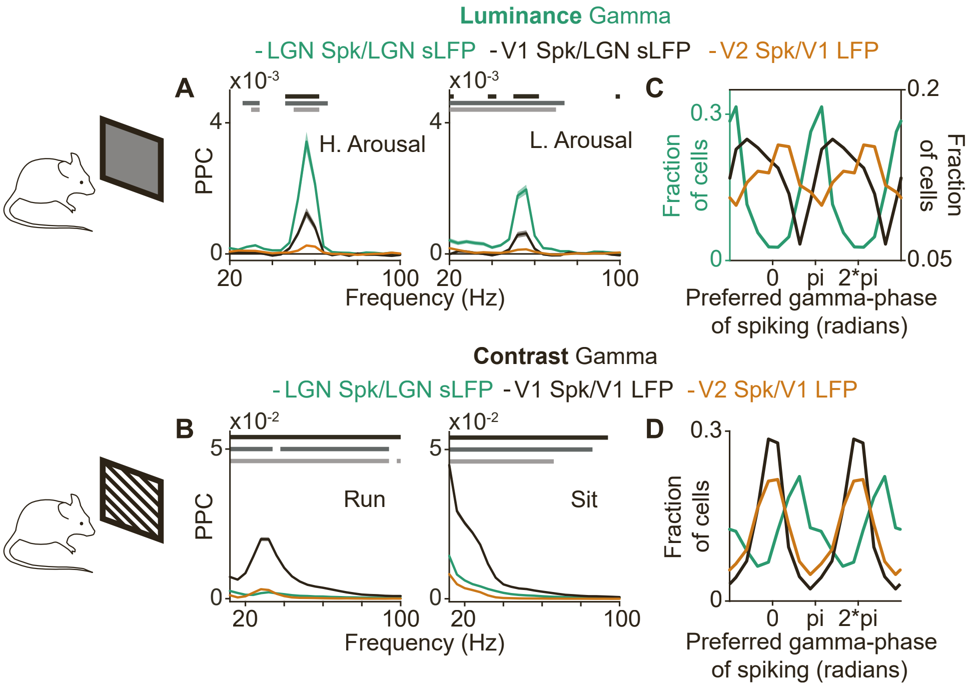

Fig. S11: Feedforward gamma-band synchronization between LGN, V1, and V2 in the mouse, and the effect of behavioral state on its strength, related to Figure 7. (A) PPC between LGN sLFPs and LGN single unit (SU) spiking (N= 2215/2119), LGN sLFPs and V1 single unit (SU) spiking (N= 2700/2917), or V1 LFPs and V2 single unit (SU) spiking (N= 11962/13121), under the luminance gamma condition. Left: Analyses based on periods of high arousal. Right: Analyses based on periods of low arousal. Bright, intermediate, and dark horizontal bars in PPC spectra designate significantly different frequency bins between the spectra of LGN SUs and V1 SUs, between the spectra of LGN SUs and V2 SUs, and between the spectra of V1 SUs and V2 SUs, respectively. **(B)** Same as A, but between V1 LFPs and LGN single unit (SU) spiking (N= 2001/2109), V1 LFPs and V1 single unit (SU) spiking (N= 2169/2435), or V1 LFPs and V2 single unit (SU) spiking (N= 7463/8387), under the grating gamma condition. Left: Analyses based on periods when the animal ran. Right: Analyses based on periods when the animal was stationary. **(A,B)** Confidence intervals designate the SEM across cells (gray rectangles designate significantly different frequency bins between populations, randomization test across cells, FDR correction for multiple comparisons with a threshold of $P< 0.05$). **(C)** Mean LGN-gamma phase of spiking for LGN (green, left y-axis) or V1 (black, right y-axis) SUs, and mean V1-gamma phase of spiking for V2 SUs (orange, right y-axis), under the condition of luminance-gamma. V2 cells lagged V1 cells, which in turn lagged LGN cells in their phase of spiking (randomization test across areas, $P< 0.05$ for all comparisons). **(D)** Mean V1-gamma phase of spiking for LGN (green), V1 (black) or V2 SUs (orange), under the condition of grating-gamma. LGN cells lagged V2 cells, which in turn lagged V1 cells in their phase of spiking (randomization test across areas, $P< 0.05$ for all comparisons).

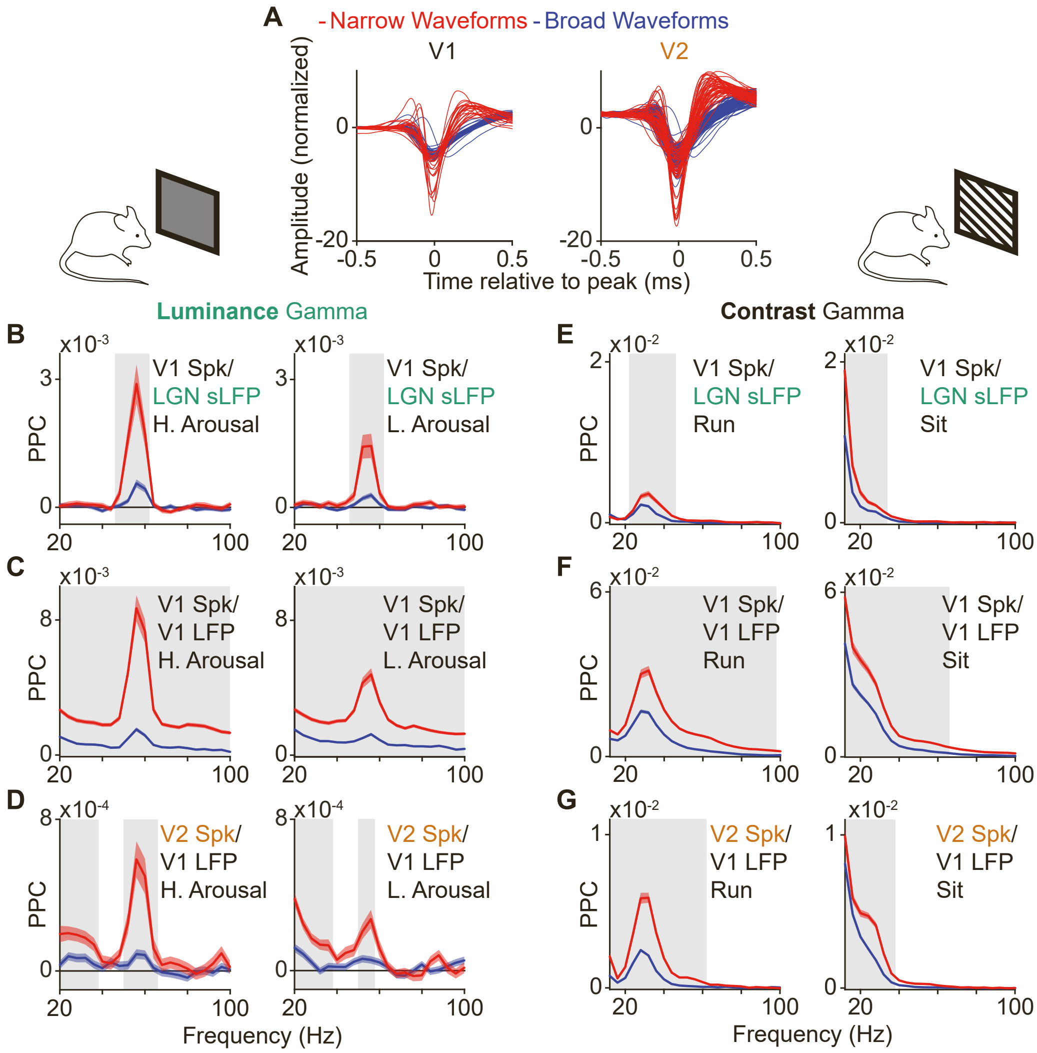

Fig. S12: The strength of intra- and inter-areal gamma-band synchronization for different cell types depends on behavioral state in the mouse, related to Figure 7. **(A)** Same as Figure 2A but for cells in mouse V1 (left) and V2 (right), for an example session. **(B)** Same as Figure 2B but for LGN sLFPs and V1 SUs, under the condition of luminance gamma (BW: N= 1913/2113, NW: N= 698/718). **(C)** Same as Figure 2B but for V1 LFPs and V1 SUs, under the condition of luminance gamma (BW: N= 2625/2950, NW: N= 928/962). **(D)** Same as Figure 2B but for V1 LFPs and V2 SUs, under the condition of luminance gamma (BW: N= 8844/9931, NW: N= 2571/2595). **(B-D)** Left: Analyses based on periods of high arousal. Right: Analyses based on periods of low arousal. **(E)** Same as Figure 2B but for LGN sLFPs and V1 SUs, under the condition of grating gamma (BW: N= 1186/1435, NW N= 476/500). **(F)** Same as Figure 2B but for V1 LFPs and V1 SUs, under the condition of grating gamma (BW: N= 1519/1747, NW: N= 582/615). **(G)** Same as Figure 2B but for V1 LFPs and V2 SUs, under the condition of grating gamma (BW: N= 5477/6091, NW N= 1585/1638). **(E-G)** Left: Analyses based on periods when the animal ran. Right: Analyses based on periods when the animal was stationary.

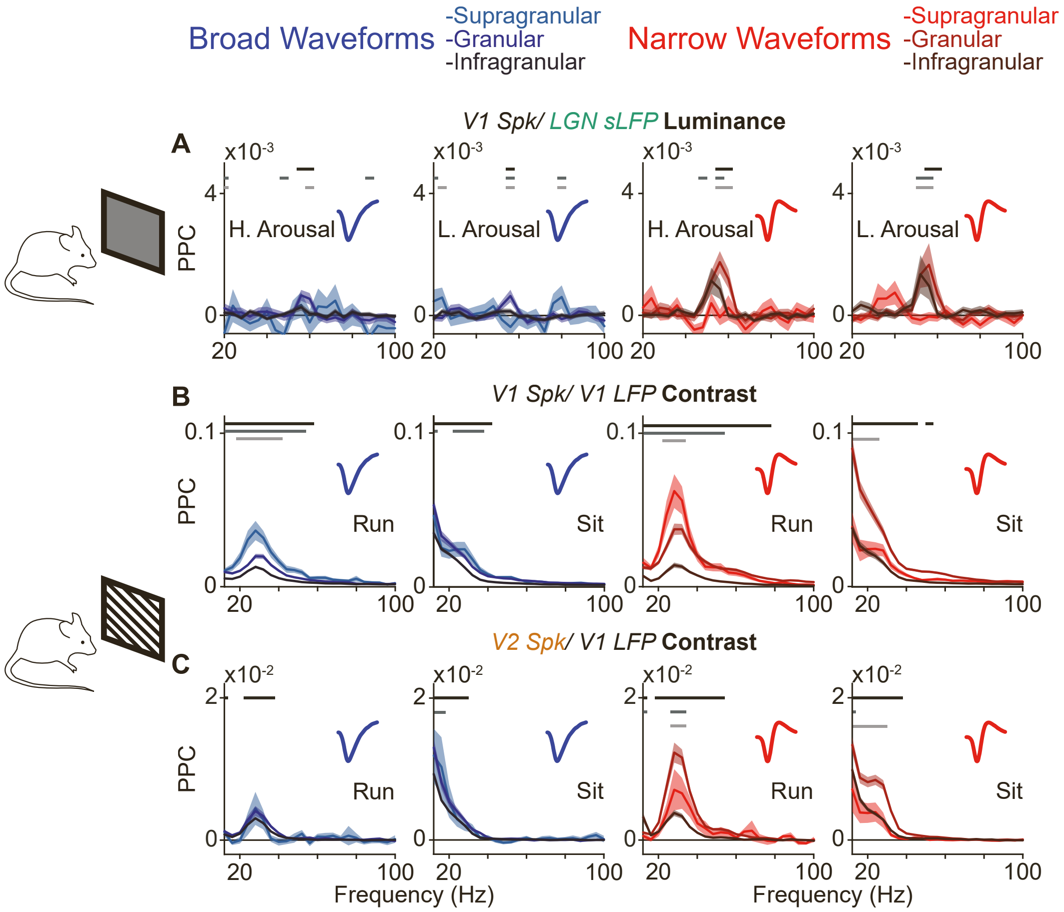

Fig. S13: Laminar patterns in the effect of behavioral state on intra- and inter-areal gamma-band synchronization in the mouse, related to Figure 7. **(A)** Same as Figure 7B but for LGN sLFPs and V1 SUs, under the condition of luminance gamma (BW: Nsup. = 50/110, Ngra. = 325/375, Ninf. = 769/774, NW: Nsup. = 41/44, Ngra. = 197/196, Ninf. = 265/259). **(B)** Same as Figure 7B but for V1 LFPs and V1 SUs, under the condition of grating gamma (BW: Nsup. = 31/32, Ngra. = 154/156, Ninf. = 197/204, NW: Nsup. = 23/55, Ngra. = 232/282, Ninf. = 556/626). **(C)** Same as Figure 7B but for V2 LFPs and V1 SUs, under the condition of grating gamma (BW: Nsup. = 23/41, Ngra. = 428/550, Ninf. = 2187/2449, NW: Nsup. = 11/15, Ngra. = 281/282, Ninf. = 544/571). **(A-C)** First and third panels from the left: Analyses based on periods when the animal ran. Second and fourth panels from the left: Analyses based on periods when the animal was stationary.

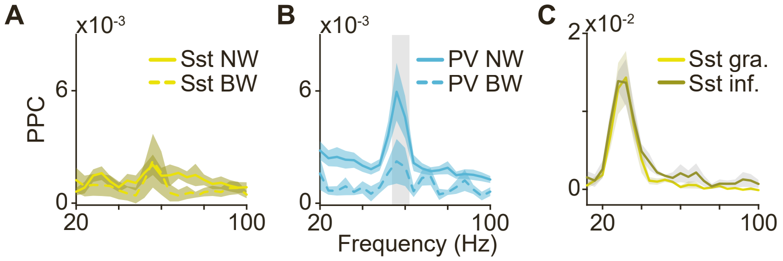

Fig. S14: Inter-areal gamma band synchronization between V1 spiking and LGN sLFPs involve NW PV+ cells, related to Figure 7. (A) PPC between V1 LFPs and V1 Sst+ cell spiking under the luminance-gamma condition, for BW (N= 19) or NW cells (N= 10). **(B)** Mean PPC spectrum between V1 LFPs and V1 PV+ cell spiking under the luminance-gamma condition, for BW (N= 9) or NW cells (N= 38). **(C)** Mean PPC spectrum between V1 LFPs and V2 Sst+ cell spiking under the luminance-gamma condition, located in the granular layer (N= 2) or the infragranular layers (N= 2). **(A-C)** Statistical comparisons are done in the same way as in Fig. 2B.
